# Novel Aspects on The Interaction Between Grapevine and *Plasmopara viticola*: Dual-RNA-Seq Analysis Highlights Gene Expression Dynamics in The Pathogen and The Plant During The Battle For Infection

**DOI:** 10.3390/genes11030261

**Published:** 2020-02-28

**Authors:** Silvia Laura Toffolatti, Gabriella De Lorenzis, Matteo Brilli, Mirko Moser, Vahid Shariati, Elahe Tavakol, Giuliana Maddalena, Alessandro Passera, Paola Casati, Massimo Pindo, Alessandro Cestaro, David Maghradze, Osvaldo Failla, Piero Attilio Bianco, Fabio Quaglino

**Affiliations:** 1 Dipartimento di Scienze Agrarie e Ambientali (DISAA), Università degli Studi di Milano, Via Celoria 2, 20133 Milano Italyalessandro.passera@unimi.it (A.P.); paola.casati@unimi.it (P.C.); osvaldo.failla@unimi.it (O.F.); piero.bianco@unimi.it (P.A.B.); fabio.quaglino@unimi.it (F.Q.); 2 Dipartimento di Bioscienze, Università degli Studi di Milano, Via Celoria 26, 20133 Milano, Italy; matteo.brilli@unimi.it; 3 Fondazione E. Mach, Centro Ricerca e Innovazione, Via E. Mach 1, 38010 San Michele all’Adige, Italy; mirko.moser@fmach.it (M.M.); massimo.pindo@fmach.it (M.P.); alessandro.cestaro@fmach.it (A.C.); 4 NIGEB Genome Center, National Institute of Genetic Engineering and Biotechnology, 14965/161 Tehran, Iran; 6002347@gmail.com; 5 Department of Crop Production and Plant Breeding, College of Agriculture, Shiraz University, 65186-71441 Shiraz, Iran; elahetavakol@gmail.com; 6 Scientific - Research Center of Agriculture, Marshal Gelovani Avenue 6, 0159 Tbilisi, Georgia; david.maghradze@gmail.com; 7 Faculty of Viticulture and Winemaking, Caucasus International University, Chargali Street 73, 0141 Tbilisi, Georgia

**Keywords:** plant–pathogen interaction, disease resistance and susceptibility, oomycete effectors, susceptibility genes, resistance genes, transcriptome, RNA-seq

## Abstract

Mgaloblishvili, a *Vitis vinifera* cultivar, exhibits unique resistance traits against *Plasmopara viticola*, the downy mildew agent. This offers the unique opportunity of exploring the molecular responses in compatible and incompatible plant-pathogen interaction. In this study, whole transcriptomes of Mgaloblishvili, Pinot noir (a *V. vinifera* susceptible cultivar), and Bianca (a resistant hybrid) leaves, inoculated and non-inoculated with the pathogen, were used to identify *P. viticola* effector-encoding genes and plant susceptibility/resistance genes. Multiple effector-encoding genes were identified in *P. viticola* transcriptome, with remarkable expression differences in relation to the inoculated grapevine cultivar. Intriguingly, five apoplastic effectors specifically associated with resistance in *V. vinifera*. Gene coexpression network analysis identified specific modules and metabolic changes occurring during infection in the three grapevine cultivars. Analysis of these data allowed, for the first time, the detection in *V. vinifera* of a putative *P. viticola* susceptibility gene, encoding a LOB domain-containing protein. Finally, the de novo assembly of Mgaloblishvili, Pinot noir, and Bianca transcriptomes and their comparison highlighted novel candidate genes that might be at the basis of the resistant phenotype. These results open the way to functional analysis studies and to new perspectives in molecular breeding of grapevine for resistance to *P. viticola*.

## 1. Introduction

The achievement of an optimal management of fungal diseases, which is effective, economically feasible for the farmer and safe for human health and the environment, requires a coordinated effort, where all the strategies available (agronomic practices, genetic selection of resistant cultivars, and biological and chemical control) are improved and integrated [1,2,3,4,5]. The deployment of resistance traits, either by conventional breeding or genetic improvement approaches, aims at obtaining crop varieties with effective resistance without impacting on other agronomically important crop traits, the quality of the product in primis [6]. A durable resistance, i.e., a resistance that remains effective while the cultivar possessing it is widely cultivated, can be achieved by pyramiding disease resistance genes in the same plant [7]. For this purpose, the identification of new resistance genes is essential for obtaining resistant varieties, which can be cultivated worldwide without being impacted by the pathogen’s adaptability and variability. Another possibility for obtaining resistant cultivars relies on the silencing of susceptibility genes, essential for compatible plant–pathogen interactions and required for successful pathogen infection [8].

The search for resistance/susceptibility traits is particularly relevant for the grapevine, a tree plant that needs to remain productive for several years while keeping its resistant features. *Vitis vinifera* L., the Eurasian grapevine species cultivated worldwide for fresh food and wine production [9], is susceptible to several pathogens, among which is the oomycete *Plasmopara viticola* (Berk. et Curt.) Berlese and De Toni. *P. viticola* causes downy mildew, one of the most severe diseases of grapes in viticultural areas with frequent rains, high humidity, and mild temperatures in the summer period [10,11]. *V. vinifera* cultivars are, generally, highly susceptible to *P. viticola*, while American and Asian species show resistant genes, associated with several Quantitative Trait Loci—QTLs [12,13]. American species such as *Vitis rupestris* Scheele, *Vitis labrusca* L., and *Vitis riparia* Mich., in particular, have been used to introgress resistant traits into *V. vinifera* since the beginning of the XIX century [14]. Currently, several grapevine varieties, like Regent in Germany and Bianca in Hungary, harboring quality traits of *V. vinifera* and resistant genes from other species, are cultivated in the open field, and several new varieties have been progressively introduced into the market [15].

Recently, the authors reported the existence of a downy mildew resistant *V. vinifera* cultivar, named Mgaloblishvili, originally from the domestication center of the species, located in Georgia (Caucasus) [16]. Experimental inoculations and microscopic investigations showed that resistance in Mgaloblishvili is associated with a significant reduction of *P. viticola* growth and sporulation compared to the susceptible *V. vinifera* cv. Pinot noir and differs from the resistance mechanism of Bianca, that is based on the hypersensitive response (programmed cell death) at the infection sites [17]. The discovery of new resistance genes belonging to the *V. vinifera* species could have a great impact on grapevine improvement, through the introgression of novel genes in already existing cultivars, containing American and Asian QTLs, or through the obtaining of pure *V. vinifera* cultivars with resistant traits. On the other hand, the identification and disruption of susceptibility genes could interfere with the host–pathogen compatibility and consequently provide disease resistance in already existing *V. vinifera* cultivars. In order to discover resistance/susceptibility traits, the mechanism underlying plant–pathogen interaction must be dissected. 

To establish pathogenesis, filamentous fungi secrete a wide repertoire of effector molecules that deregulate the plant immune responses and facilitate host colonization [18]. The plant defense system is first activated upon recognition of pathogen-associated molecular patterns (PAMPs) through transmembrane receptors, called pattern recognition receptors (PRRs), leading to the secretion of antifungal enzymes at the apoplastic site and PAMP Triggered Immunity (PTI). Successful pathogens interfere with the host’s immune system by delivering cytoplasmic effectors inside the plant cell and secreting apoplastic effectors into the plant extracellular space [19], leading to susceptibility (Effector Triggered Susceptibility—ETS). Effectors are pathogen-encoded proteins that lack of clear sequence similarity to known function, do not always have enzymatic activity and possess, in most cases, a high sequence diversity [20]. Among the known cytoplasmic effectors, encoded by genes expressed by *P. viticola*, are the RXLR, CRN, and YxSLK classes [21,22,23,24,25,26,27]. The RXLR class of effectors is defined by a conserved N-terminal RXLR motif and a diverse C-terminal domain, responsible for the effector activity inside the host cell [20]. The CRNs (“Crinkler”) effectors are widespread across the oomycete lineage, and can cause cell death in the host plant in some cases [28,29]. They consist of modular factors that are secreted and translocated inside host cells by means of a conserved N-terminal domain, with a characteristic LXLFLAK motif, and execute their presumed effector function in the host nucleus through the C-terminal domain [29]. The YxSL[RK] proteins, first discovered in *Pythium ultimum* Trow [30] and recently found in *P. viticola* [21], show a modular organization with a conserved amino-terminal region with four conserved motifs and a highly variable C-terminus that make them candidates to be considered as an effector family. Oomycete apoplastic effectors include a large number of hydrolytic enzymes, which are involved in the degradation of host cell components, enabling penetration of host cells [31]. Apoplastic effectors act at the extracellular side by inhibiting the activity of antifungal enzymes, such as proteases and 1,3-glucanases, produced by the plant [32] or can be translocated inside the host cell, through a poorly understood mechanism [33], that can be mediated by haustoria as in the case of EPIC1 in *Phytophthora infestans* (Mont.) de Bary [34]. Apoplastic effectors known in the *P. viticola* group include trypsin, elicitin, and NPP1 effectors [21,27]. Serine proteases and specifically trypsin-like enzymes are involved in the response mechanism of oomycetes against plant defenses [35]. Elicitins are structurally conserved extracellular proteins in *Phytophthora* and *Pythium* species known to sequester sterols from the host plant, to overcome their inability to synthesize these lipids [31]. NPP1 effectors belong to the necrosis-inducing proteins, known as Nep1-like proteins or necrosis- and ethylene-inducing peptide 1-like proteins (NLPs) [20,36]. In addition to the above-cited effectors, proteins possessing a signal peptide for secretion could putatively act as effectors themselves [20].

Effector recognition by the host may occur through direct effector–receptor binding or by sensing the perturbing activity of an effector on host components [37]. Effectors delivered to the host cytoplasm can be recognized by intracellular resistance (R) proteins of the cytoplasmic nucleotide binding site leucine-rich repeat (NBS-LRR) class [38] leading to immunity (Effector Triggered Immunity—ETI). The pathogen growth can be blocked through programmed cell death (hypersensitive response) at the infection site, as occurs in American grapevine species, or by the synthesis of antifungal compounds and structural barriers mediated by a complex response pathway, that involves hormones such as salicylic acid, jasmonic acid, and ethylene [39], as occurs in Mgaloblishvili [17].

To get insight into the resistance mechanism of Mgaloblishvili, a comparative transcriptomic study, where the differentially expressed genes from Mgaloblishvili leaves inoculated or non-inoculated with *P. viticola* were compared to those of Pinot noir (susceptible control) and Bianca (resistant control harboring *Rpv3* QTL of American origin), was undertaken. Since the genomes of Mgaloblishvili and Bianca are still not available, the reads were mapped against predicted mRNAs of a Pinot noir selfing reference transcriptome (PN40024 12X v2). Transcriptomic analysis led to the discovery of unique resistant traits of Mgaloblishvili associated with the overexpression of genes related to pathogen recognition, the ethylene signaling pathway, synthesis of antimicrobial compounds and enzymes, and development of structural barriers [17].

In this study, we used the same transcriptomic data to further dissect the molecular mechanisms underlying the interaction between *P. viticola* and grapevine. Based on the analysis of *P. viticola* transcriptome on Mgaloblishvili, Pinot noir, and Bianca, we first aimed at identifying the pathogen genes and pathways involved in the interaction with resistant (Mgaloblishvili and Bianca) and susceptible (Pinot noir) grapevines. Then, we explored the pathways involved in susceptibility or resistance to the pathogen based on network analysis of Mgaloblishvili, Pinot noir, and Bianca transcriptomes. Finally, we performed a de novo assembly of the grapevines transcriptomes to discover novel transcripts, not present in the reference genome, that might be at the basis of the resistant phenotype.

## 2. Materials and Methods

### 2.1. RNA-seq Data 

Whole transcriptomes of leaves collected from Mgaloblishvili (M), Pinot noir (P), and Bianca (B) plants inoculated and non-inoculated with *P. viticola* at 0, 1, 2, and 3 days after inoculation (dai) published in [17] (Study: PRJEB24540; European Nucleotide Archive) were used to i) analyze the *P. viticola* transcriptome; ii) analyze grapevine coexpression gene patterns; and iii) perform de novo transcriptome assembly.

### 2.2. P. viticola Transcriptome: Read Preparation, Alignment, and Analysis of Differentially Expressed Genes

After checking read qualities using FastQC1, trimming was performed with the following Trimmomatic2 command:

java-jar trimmomatic-0.36.jar PE R1.fastq R2.fastq \\

HEADCROP:20 SLIDINGWINDOW:4:15 MINLEN:30

Only pairs for which both mates survived the above command were further used. Here, Rn.fastq indicates first and second mate file.

Alignments against the *P. viticola* genome were performed with the following Tophat command:

tophat-G PVIT_GENES_v2.gff3 --segment-length 17 --transcriptome-index \\

PVITGENESv2 -i 20 -I 100,000 -g 10 -p 3 --library-type fr-firststrand \\

PVITv2 R1_trimm.fastq R2_trimm.fastq 

where PVIT_GENES_v2.gff3 contains all the predicted genes in the *P. viticola* genome and PVITv2 the genome sequence for the strain sequenced in [21] and are both available at the *Plasmopara viticola* multi omics dissection project [40]. Here, R1_trimm.fastq R2_trimm.fastq indicates the mates after trimming. Alignments were then filtered to retain only unique mapping reads, by exploiting the flag NH:i:1 that indicates alignments involving reads with only one alignment.

Transcript abundance was quantified by using htseq-count and by setting -s reverse to take into account the correct strandedness of the libraries.

Analysis of differentially expressed genes (DEGs) was performed only for the 3 dai time point, where there is the largest number of reads mapping on the *P. viticola* genome, for each variety; for this task we considered only genes with at least 20 reads in total over the 9 libraries at 3 dai. We did not apply a more stringent threshold as DESeq2 itself will remove low counts genes; in DESeq2 we used the following thresholds: alpha = 0.1, minimum absolute value of the log2 fold change (LCF) = 1, therefore we will report only differentially expressed genes that have an absolute change of their expression level across two samples of at least 2-fold.

### 2.3. GO Enrichments for P. viticola DEGs

It was evaluated whether the set of DEGs from specific functional categories are enriched in the different samples by using the GO (Gene Ontology) annotation of *P. viticola* [21] and the webserver AgriGO [41]. Enriched biological processes from this analysis were then reduced by exploiting ReviGO [42] and taking into account only GO processes with a *p*-value for enrichment smaller than 1E-03.

The annotation of cytoplasmic and apoplastic effectors and genes having a signal peptide for secretion made in [21] was intersected with the differentially expressed genes from this work to identify the transcripts differentially expressed by *P. viticola* when infecting Mgaloblishvili and Pinot noir (PvM_*vs*_PvP), Mgaloblishvili and Bianca (PvM_*vs*_PvB), and Pinot noir and Bianca (PvP_*vs*_PvB). Three-way Venn diagrams showing the overlap among different contrasts were built using the jVenn web-server [43], using DEG lists as input data. The function heatmap.2 of gplots R package [44] was used for the graphical representation of gene expression and hierarchical clustering.

### 2.4. Network Analysis of Grapevine Transcriptome: Gene Coexpression Network Analysis

Matrix of raw count data of gene expression including all replicates was imported to DESeq2 [45] and a model.matrix was defined according groups. After normalization by library size and RLE (Relative Log Expression) methods a matrix of average by group was prepared in DESeq2. After filtering out the low expressed genes, resulted combined gene expression matrix was used for gene coexpression network analysis using the log2-transformed expression values of the most variable genes (3000) and the WGCNA package v1.51 [46]. Briefly, a softpower β was chosen using the function pickSoftThreshold to fit the signed network to a scale-free topology. Next, an adjacency matrix was generated. Topological Overlap Matrix (TOM) was used as an input in the function hclust (“average” method) to construct a hierarchical clustering tree (dendrogram). TOM is a measure of topological similarity between the genes within a network, i.e., it evaluates whether two or more nodes share links within the network and groups them into the same module [47,48]. A threshold of 0.15 (correlation > 85%) was chosen to merge similar modules, and only modules having at least 20 genes were kept. 

### 2.5. Network Analysis of Grapevine Transcriptome: Functional Enrichment Analyses 

GO and pathway enrichment analyses of gene sets (within networks or modules) were performed by in-house scripts using GOseq and KEGG as the data sources. *p*-values were determined through a Fisher Exact test and adjusted via the Benjamini–Hochberg’s method. A threshold of adjusted *p*-value < 0.05 was used to determine the statistical significance of enrichment results.

### 2.6. Network Analysis of Grapevine Transcriptome: Transcription Factor Enrichment

To identify potential common transcription factors (TFs) that may control transcription of module genes, the promoter region of the genes of each module were investigated for transcription factor binding sites (TFBS) using Plant TFDB database and TF enrichment tools and a hypergeometric test was applied to enrich the statistically significant elements and related TFs (*p*-value ≤ 0.05). *p*-values were adjusted for multiple testing corrections using Benjamini and Hochberg’s false discovery rate (FDR) method (*p* ≤ 0.05).

### 2.7. De novo Transcriptome Assembly of Grapevine Cultivars

The raw RNA-seq datasets for the 63 paired ends (PE50) libraries available for Pinot noir (P), Bianca (B), and Mgaloblishvili (M) were retrieved and prepared for a de novo assembly approach. For each genotype, three biological replicates were available for the non-inoculated plants (ni) at zero dai (ni0dai; untreated control plants), day one (ni1dai), day two (ni2dai), and day three (ni3dai), and the inoculated plants (i) at day one (i1dai), day two (i2dai), and day three (i3dai). The triplicate libraries of each sample were merged in one single dataset, respectively, but keeping right and left reads into two separate files. The merged datasets were used for the de novo assembly procedure performed with Trinity [49]. The parameters used were --seqType fq --max_memory 60G --left --right --include_supertranscripts --SS_lib_type RF --CPU 4 --trimmomatic. The assemblies were run on a HPC cluster [50].

The de novo assembled transcriptomes were then merged in a single dataset and a clustering was performed using cd-hit-est (c = 0.95) [51]. The clusters formed only by transcripts belonging to either Bi, Bni, Mi, Mni, or combinations of these but not clustering with Pi or Pni were selected. Similarly, clusters formed only by Pi or Pni or combinations of these were retrieved separately. The cluster consensus sequences with a length ≥ 300 bp were retrieved obtaining a dataset of transcripts for each genotype. These transcripts were then subjected to blastx (evalue 1E-7) against a dataset formed by all the plant proteins having a UniProt database entry.

The blastx outputs were filtered, considering only the hits having a ratio (alignment length/subject length) ≥ 0.8 and pident ≥ 70.00. The UniProt IDs of the retrieved blastx hits for B and M were compared with the IDs retrieved for Pi/Pni for both the inoculated and non-inoculated state, to find unique IDs characterizing Bi, Bni, Mi, Mni, Pi, and Pni. The sequences corresponding to the transcripts associated with the IDs obtained were subjected to Gene Ontology and enrichment analyses using the Blast2GO suite (version 5.2.5) [52].

### 2.8. Statistical Analysis of Differentially Expressed De novo Transcripts

The de novo transcriptomes generated for each genotype and condition were clustered separately using cd-hit-est with the parameter c = 0.95. The RNA-seq reads passing the quality filter set in the Trinity step and used to assembly the transcriptome de novo were re-aligned on their respective clustered transcriptome. Bowtie2 (version 2.3.4.3) [53] was used to align reads with the default parameters and -L 31. The read count number aligning on each transcript was calculated using an in-house script counting the alignments having the SAM flag 99/147, 83/163, 81/161, and 97/145. A read count matrix was generated and used for the subsequent analysis. DESeq2 R package [45] was used to perform DEG analysis, by a multifactor design method, among the different treatments (non-inoculated vs. inoculated) of each variety at each time point. Transcripts with more than 5 reads were retained for the analysis. For each transcript, the log2 fold change (FC), *p*-value and adjusted *p*-value were evaluated, and only transcript IDs with false discovery rate (FDR)-adjusted *p*-value < 0.1 were retained. Three-way Venn diagrams showing the overlaps among different time points per genotype were built using the jVenn web-server [43], using DEG lists as input data. 

### 2.9. Availability of Data and Materials

The datasets for this study can be found in the European Nucleotide Archive (ENA) under the study accession number: PRJEB24540. 

## 3. Results

### 3.1. P. viticola Transcriptome Analysis

The analysis of *P. viticola* transcriptome on Mgaloblishvili (PvM), Pinot noir (PvP), and Bianca (PvB) showed that 5251 genes were expressed by the pathogen at 3 dai. Further analysis highlighted the presence of 1519 DEGs when infecting Mgaloblishvili in comparison with Pinot noir (PvM_*vs*_PvP), Mgaloblishvili in comparison with Bianca (PvM_*vs*_PvB), and Pinot noir in comparison with Bianca (PvP_*vs*_PvB; Table 1). Only two genes were differentially expressed by *P. viticola* during infection on Mgaloblishvili with respect to Pinot noir (PvM_*vs*_PvP), while 533 and 984 genes were differentially expressed by the pathogen when comparing the inoculation of Mgaloblishvili and Bianca (PvM_*vs*_PvB) and Pinot noir and Bianca (PvP_*vs*_PvB), respectively (Table 1). *P. viticola* DEGs were mostly up-regulated (96%) in Mgaloblishvili and Pinot noir with respect to Bianca (Table 1; Table S1).

**Figure 1:**
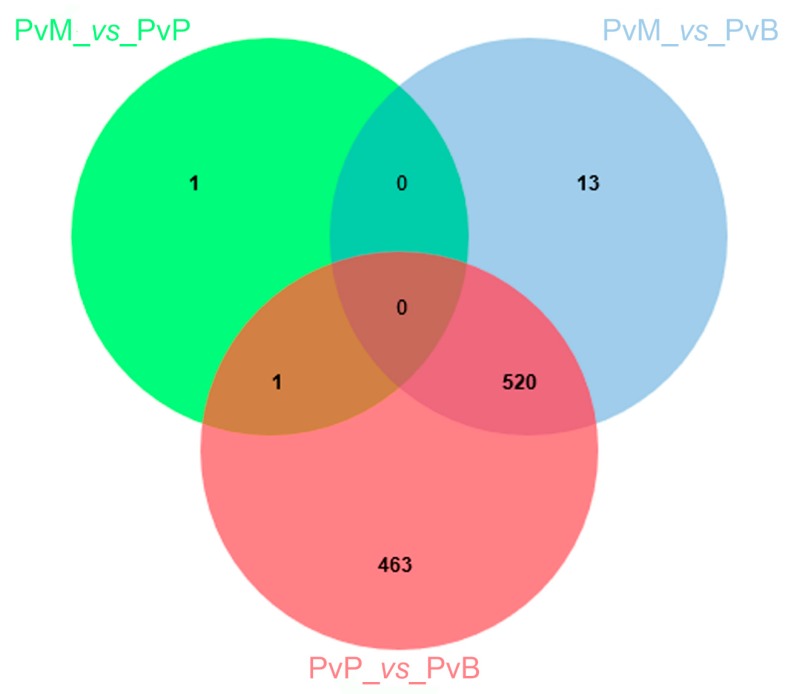
Venn diagram illustrating the comparison between genes differentially expressed (DEGs) by *Plasmopara viticola* (Pv) following inoculation on Mgaloblishvili (M) and Pinot noir (P) (PvM_*vs*_PvP), M and Bianca (B) (PvM_*vs*_PvB), and P and B (PvP_*vs*_PvB).

To identify how the *P. viticola* transcriptome is modulated upon infection of grapevine accessions with different susceptibility levels, we mainly focused on *P. viticola* DEGs specific for each contrast (PvM_*vs*_PvP, PvM_*vs*_PvB, and PvP_*vs*_PvB) or shared among contrasts (PvM_*vs*_PvP with PvP_*vs*_PvB; PvM_*vs*_PvB with PvP_*vs*_PvB; PvM_*vs*_PvP with PvM_*vs*_PvB; and PvM_*vs*_PvP with PvP_*vs*_PvB and PvM_*vs*_PvB). The number of DEGs within and among contrasts is reported in Figure 1. Two *P. viticola* DEGs were found in the comparison between Mgaloblishvili and Pinot noir (PvM_*vs*_PvP), one specific and one shared in the contrast Pinot noir/Bianca (PvP_*vs*_PvB). The comparison of *P. viticola* transcriptome on Mgaloblishvili and Bianca (PvM_*vs*_PvB) and Pinot noir and Bianca (PvP_*vs*_PvB) resulted in the presence of 13 and 463 specific DEGs, respectively. Five hundred and twenty DEGs were common in the comparisons Mgaloblishvili/Bianca and Pinot noir/Bianca (PvM_*vs*_PvB with PvP_*vs*_PvB), while no DEGs were shared among the three contrasts (PvM_*vs*_PvP with PvP_*vs*_PvB and PvM_*vs*_PvB) nor between PvM_*vs*_PvP with PvM_*vs*_PvB. 

Among the most over-represented *P. viticola* biological processes (frequency higher than 4%), shared in PvM_*vs*_PvB and PvP_*vs*_PvB contrasts, there are metabolic processes related to “nitrogen compounds” (GO:0034641), “proteins” (GO:0019538), and “small molecules” (GO:0044281), “biosynthetic processes” (GO:0009058 and GO:0044249), “gene expression” (GO:0010467), “oxidation–reduction process” (GO:0055114), and “response to stimuli” (GO:0050896; Table S2). Differences between biological processes over-represented in the two contrasts are mainly related to “response to stress” (GO:0006950) in PvM_*vs*_PvB, which was not represented in PvP_*vs*_PvB.

### 3.2. P. viticola Effectors

**Figure 2:**
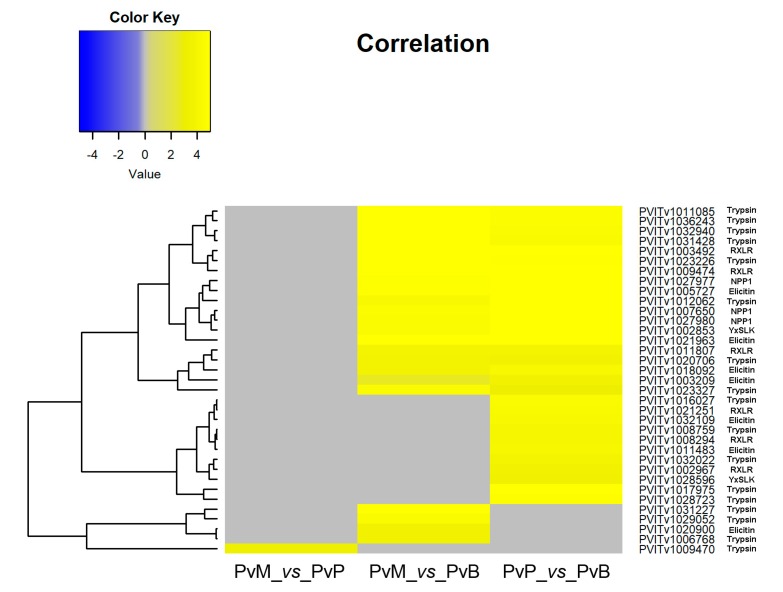
Heatmap of 35 genes encoding cytoplasmic (RXLR and YxSLK) and apoplastic effectors (trypsin, elicitin and NPP1) differentially expressed by *Plasmopara viticola* (Pv) following inoculation on Mgaloblishvili (M) and Pinot noir (P) (PvM_*vs*_PvP), M and Bianca (B) (PvM_*vs*_PvB), and P and B (PvP_*vs*_PvB).

**Figure 3:**
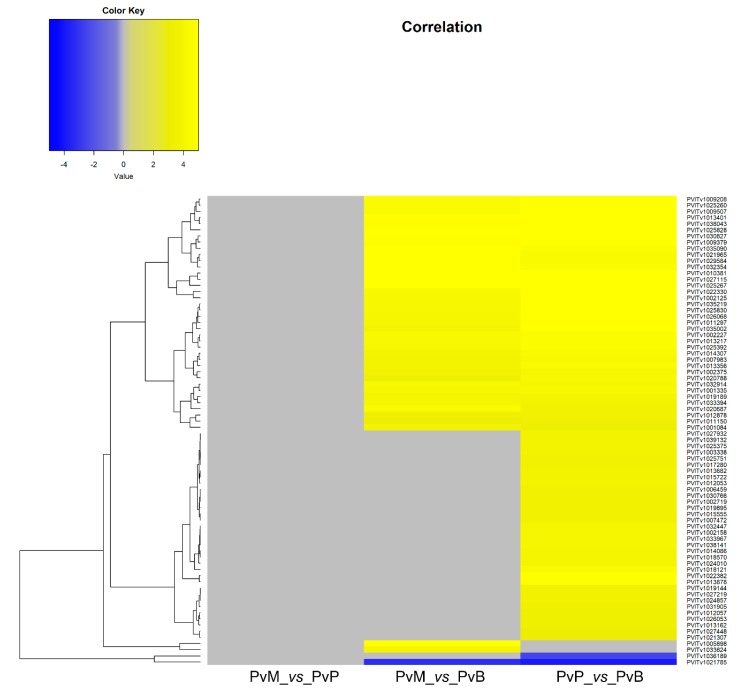
Heatmap of 76 genes encoding effectors possessing a signal peptide for secretion differentially expressed by *Plasmopara viticola* (Pv) following inoculation on Mgaloblishvili (M) and Pinot noir (P) (PvM_*vs*_PvP), M and Bianca (B) (PvM_*vs*_PvB), and P and B (PvP_*vs*_PvB).

The transcripts encoding for proteins with known or putative *P. viticola* effector function were identified among the *P. viticola* DEGs at 3 dai. Thirty-five cytoplasmic and apoplastic effectors and seventy-six *P. viticola* genes encoding proteins possessing a signal peptide for secretion were differentially expressed by *P. viticola* when comparing the pathogen transcriptomes on Mgaloblishvili and Pinot noir (PvM_*vs*_PvP), Mgaloblishvili and Bianca (PvM_*vs*_PvB), and Pinot noir and Bianca (PvP_*vs*_PvB; Table S3; Figure 2; Figure 3). A single *P. viticola* gene (PVITv1009470), encoding a trypsin-like apoplastic effector, was differentially expressed only during the interaction with Mgaloblishvili compared to Pinot noir (PvM_*vs*_PvP; Figure 2). Four *P. viticola* genes encoding apoplastic effectors (the elicitin PVITv1020900 and trypsin PVITv1006768, PVITv1031227, and PVITv1029052; Figure 2) and two genes encoding proteins possessing a signal peptide for secretion (PVITv1033624, and PVITv1005898; Figure 3) were up-regulated only in Mgaloblishvili compared to Bianca (PvM_*vs*_PvB). In general, none of the transcripts associated with elicitin and NPP1 effectors showed any known function. PVITv1033624 and PVITv1005898 encoded enzymes involved in β-glucan hydrolysis (glucanendo-1,6-beta-glucosidase) and pectin de-esterification (pectinesterase).

Fifty-seven transcripts, corresponding to four cytoplasmic effectors (three RXLR: PVITv1011807, PVITv1009474, PVITv1003492; and one YXSLK: PVITv1002853), fifteen apoplastic effectors (from PVITv1021963 to PVITv1031428), and 38 genes encoding secreted proteins (from PVITv1011150 to PVITv1002125), were shared by the pathogen when infecting both *V. vinifera* cultivars compared to Bianca (PvM_*vs*_PvB and PvP_*vs*_PvB). The cytoplasmic effectors do not possess any known function apart from PVITv1011807, which has a nucleotide binding function, and PVITv1009474 that encodes tetrahydrodipicolinate N-acetyltransferase. Among the genes with signal peptide for secretion, some encoded enzymes involved in host cell wall degradation (xylan1,4-beta-xylosidase, glucan1,3-beta-glucosidase, cellulase, cellulose1,4-beta-cellobiosidase non-reducing end, and pectinesterase), protein modification (protein disulfide-isomerase, peptidylprolyl isomerase, and dolichyl-diphosphooligosaccharide-protein glycotransferase), transport (proton-exporting ATPase and adenosinetriphosphatase), and nutrition (6(G)-fructosyltransferase).

Forty-five transcripts, corresponding to four cytoplasmic effectors (three RxLR: PVITv1002967, PVITv1021251, PVITv1008294; and one YxSLK: PVITv1028596), seven apoplastic effectors (from PVITv1011483 to PVITv1028723), and 34 transcripts coding for secreted proteins (from PVITv1025751 to PVITv1038141), were specifically associated with the pathogen when infecting the *V. vinifera* cv. Pinot noir in the comparison with Bianca (PvP_*vs*_PvB). The YxSLK effector encodes a spermidine synthase, while the others are completely uncharacterized, as none of them have significant homology with characterized proteins. Among the genes encoding for secreted proteins, some were linked to oxidative stress (6-phosphogluconolactonase, monodehydroascorbate reductase NADH) and nutrition (acid phosphatase).

The above cited genes were all up-regulated with a LFC (log2 Fold Change) greater than 3. Only two effectors (PVITv1021785 and PVITv1036189), encoding uncharacterized proteins, were down-regulated in the two *V. vinifera* varieties compared to Bianca (PvM_*vs*_PvB and PvP_*vs*_PvB).

### 3.3. Grapevine Transcriptome Network Analysis

**Figure 4:**
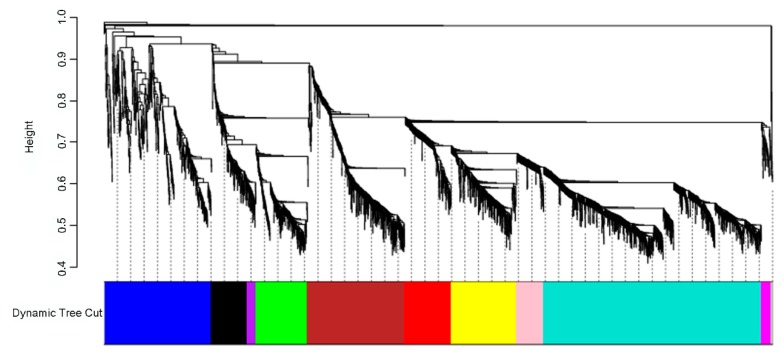
Identification of gene coexpression modules of all samples data (24 samples, 3000 genes) using average hierarchical linkage clustering; the *y*-axis denotes the coexpression distance and the *x*-axis corresponds to genes. Dynamic tree cutting was applied to identify modules by dividing the dendrogram at significant branch points. Modules are displayed with different colors in the horizontal bar.

The network analysis was performed on 3000 genes with the highest gene expression variation across samples. Genes with similar expression pattern were grouped into modules. A total of 10 coexpression modules were identified via hierarchical clustering (Figure 4). 

The number of genes per module (module size) ranged from 40 (purple) to 977 (turquoise). Gene Set Enrichment analyses were performed on the modules to identify enriched GO terms or pathways (Table S4), showing that all the modules, except the purple one, had significant enrichments, providing evidence of functional coherence within modules. Some of the most highly significant GO terms and KEGG pathways were: “monoterpenoid biosynthesis” and “glutathione metabolism” (green module); “*alpha*-linolenic acid metabolism”, “biosynthesis of secondary metabolites”, and “flavonoid biosynthesis” (black module); “glucosinolate biosynthesis”, “cyanoamino acid metabolism”, and “plant–pathogen interaction” (blue module); “flavone and flavonol biosynthesis” and “phagosome and cutin, suberine, and wax biosynthesis” (brown module); “linoleic acid metabolism” (pink module); “plant hormone signal transduction” (red module); “phenylpropanoid biosynthesis” and “flavonoid biosynthesis” (turquoise module); and “plant–pathogen interaction” and “monoterpenoid biosynthesis” (yellow module).

All genes of each module were screened to identify transcription factors as main regulators of modules: turquoise module includes the most TFs (Table S5). In addition, the analysis of the conserved Transcription Factor Binding Sites (TFBSs) on promoter regions of genes included in each module revealed that a total of 299 TFBSs (*p*-value < 0.05) were over-represented across modules, except in the purple and magenta modules. Each module was enriched in minimum 14 (green module) and maximum 89 (blue module) TFBSs (Table S6). These findings indicated that WGCNA software could efficiently exploit gene expression levels to build modules able to recover coregulation.

Gene expression pattern for each module was extracted from the network analysis to determine the genes discriminating for resistance/susceptibility to *P. viticola*. Only the yellow and the blue module genes were selected for patterning since they included most of the genes involved in the plant–pathogen interaction pathways (Table S4): the yellow module mainly associated with resistance to *P. viticola* in Bianca, while the blue module related to the response of Mgaloblishvili and Bianca to the pathogen. In the yellow module, five main clusters have been identified and three of them showed a different expression pattern among cultivars. Mgaloblishvili and Pinot noir showed similar expression patterns, different from those of Bianca (where the majority of genes were up-regulated; Figure S1). This module included key genes that mimic Bianca response to the pathogen, such as genes encoding for receptor kinase proteins, PR proteins, or genes related to the signaling pathway, production of ROS (Reactive Oxygen Species) and antimicrobial compounds. In the blue module, four main clusters have been identified. Most of them showed a similar expression pattern among the three cultivars (mainly up-regulated), other showed a similar expression pattern between Bianca and Pinot noir (mainly down-regulated), different from the expression pattern of Mgaloblishvili (Figure S2). This module included genes related to unique Mgaloblishvili resistant behavior, such as genes encoding for protein ubiquitination, mitogen-activated protein kinases, wall-associated receptor kinases, cellulose, and lignin biosynthesis proteins.

### 3.4. De novo Grapevine Transcriptome Assembly

Table 2 summarizes the characteristics of the libraries used for each genotype (M, Mgaloblishvili; P, Pinot Noir; B, Bianca) and treatment (i = inoculated; ni = non-inoculated). The raw reads ranged from 5,042,556 to 35,478,918 with an average per triplicate ranging from 20,481,001 (Mni at 3 dai) to 29,590,959 (Mi at 3 dai). The reads available for the de novo transcriptome assembly that passed the trimmomatic filtering step ranged from 59,610,375 (Mni at 3 dai) to 86,910,171 (Mi at 3 dai). In total, 21 de novo transcriptomes were assembled (Table 2). The number of transcripts was comprised between 38,749 (Pni at 0 dai) and 60,459 (Bi at 3 dai) whereas the number of supertranscripts ranged between 28,818 (Pni at 0 dai) and 37,891 (Bi at 3 dai). 

The transcriptomes were merged and clustered using cd-hit-est producing 171,743 clusters. A selection on the clusters was carried out to retrieve only those formed by transcripts belonging to a single genotype at the three time-points together.

The selection resulted in 30,201 clusters for Mi and/or Mni, 26,756 clusters for Pi and/or Pni, and 50,515 clusters for Bi and/or Bni. In addition, clusters containing transcripts belonging to Mi, Mni, Bi, and Bni but not Pi and/or Pni were also selected resulting in 47,020 clusters. Subsequently, the sequences of each selected cluster subset were filtered retrieving only those with a length ≥300 bp to be used in the blastx analysis. The transcripts obtained in each subset were: 16,225 for Mi/Mni, 14,000 for Pi/Pni, and 28,185 for the Bi/Bni. Moreover, 12,579 and 17,258 transcripts were retrieved for Mi/Mni and Bi/Bni, respectively, from clusters formed by transcripts of Mi/Mni and Bi/Bni in any combination.

This analysis focused on differences at nucleotide level and length between the transcripts assembled for each genotype. The aim was to highlight those transcripts differing among the transcriptomes that could be considered as typical and characteristic (unique) for each genotype investigated. However, transcripts differing at nucleotide level with the parameters used in our analysis could have higher homology at protein level. Therefore, a blastx analysis was performed and the Uniprot IDs (IDs) of the hits with pident ≥70.00 and a ratio alignment/subject length ≥0.8 were extracted separately for each genotype and treatment (inoculated or non-inoculated). The number of uniprot IDs was: 1802 associated with Mi transcripts, 2526 with Mni, 525 with Pi, 959 with Pni, 2477 with Bi, and 3076 with Bni. Further, to identify proteins unique for M and B genotypes, the IDs of Mi, Mni, Bi, and Bni were compared to those of Pni and Pni together obtaining 1186 for Mi, 1590 for Mni1, 786 IDs for Bi, and 2183 for Bni. In addition, comparison between the unique IDs for Mi and those unique for Mni resulted in 510 Uniprot IDs characterizing Mi and 803 characterizing Mni. The number of IDs unique for Pi and Pni was 136 and 291, respectively. Similarly, the ID found uniquely for Bi were 625 whereas for Bni were 867. 

Gene Ontology and enrichment analyses were carried out to identify the most enriched GO categories in inoculated *vs* non-inoculated samples per cultivar (Table S7, Figures S3–S5). Based on the biological process GO categories, the most enriched terms were “phosphorylation” and “oxidation–reduction” for Mgaloblishvili (16% and 14% respectively) and Bianca (15% and 16% respectively); and “cellular protein modification” and “oxidation–reduction” for Pinot noir (16% and 12% respectively). The most enriched cellular component GO category was “integral component of membrane” per cultivar. While, about the molecular function GO categories, Mgaloblishvili showed an enrichment of “nucleic acid binding” terms (14%), Pinot noir an enrichment of “protein binding” terms (22%), and Bianca inoculated samples “hydrolase activity” (18%).

### 3.5. Unique Gene Expression Patterns of Grapevine Cultivars Under Infection

In Table 3, an overview of DEGs per each cultivar at each time point, obtained by analyzing their de novo assembly, is reported. At 1 dai, Bianca showed the highest number of DEGs (around 16,300) and Pinot noir the lowest (around 2700). At 2 dai, Pinot noir showed the highest number of DEGs (around 900) and Mgaloblishvili the lowest (around 200). At 3 dai, Bianca showed the highest number of DEGs (around 11,000) and Mgaloblishvili the lowest (160). The list of DEGs for Mgaloblishvili, Pinot noir, and Bianca are reported in the Tables S8–S10. An analogous number of up and down regulated DEGs was observed for each cultivar and time point. The time point showing the lowest percentage of DEGs for each cultivar was 2 dai. The highest number of shared DEGs per cultivar was detected among 1 and 3 dai (Figures S6–S8). All the combinations showed DEGs in common among the three time points within each variety. For each variety, only a low percentage of DEGs were unique transcripts. For Mgaloblishvili at 1 dai, 85 out of 6568 (1.3%) DEGs were identified as unique transcripts, of which 27 were up-regulated and 58 down-regulated. The ones passing the LFC threshold were: a receptor-like serine/threonine-protein kinase SD1-7 (isoform X1), an APK1A protein kinase, two 18.3 kDa heat shock proteins (class I), and a valencene synthase (VvVal), all up-regulated; and a probable xyloglucan endotransglucosylase/hydrolase protein, a putative 4-hydroxy-4-methyl-2-oxoglutarate aldolase, a delta(24)-sterol reductase, a cytochrome P450 CYP736A12, and a hypothetical protein VITISV_031197, all down-regulated. For Pinot noir at 1 dai, 15 out of 2698 (0.6%) DEGs were identified as unique transcripts. The ones passing the LFC threshold were: a cytokinin riboside 5’-monophosphate phosphoribohydrolase (LOG8), a low temperature-induced protein (lt101.2), and an unnamed protein product, all up-regulated; and two hypothetical proteins (VITISV_031197 and VITISV_038036), all down-regulated. For Bianca at 1 dai, 277 out of 16,448 (1.7%) DEGs were identified as unique transcripts. The ones passing the LFC threshold were a terpene synthase, a LON peptidase N-terminal domain and RING finger protein, a protein STAY-GREEN 1, and a chloroplastic probable calcium-binding protein CML20, all up-regulated; and a putative glycerol-3-phosphate transporter 1, an elongation factor 1-alpha, an unnamed protein product, a partial alpha-ketoglutarate-dependent dioxygenase, a NADH dehydrogenase subunit 3, two 17.3 kDa small heat shock protein, a flavonoid 3’,5’-hydroxylase 2, an auxin transporter-like protein 3, a thiamine biosynthetic bifunctional enzyme (isoform X1), a NADH dehydrogenase subunit 7, a Shaggy-related protein kinase alpha, a probable inactive ATP-dependent zinc metalloprotease FTSHI 4, a chloroplastic LINE-1 reverse transcriptase-like, and a PSII 32 kDa protein, all down-regulated.

## 4. Discussion

### 4.1. P. viticola Transcriptome

In a previous study [17], the authors showed that Mgaloblishvili, a Georgian (Caucasus) cultivar of *V. vinifera,* is able to reduce growth and sporulation of *P. viticola,* compared to the susceptible *V. vinifera* control (Pinot noir), by four and ten times, respectively. In contrast, the interspecific hybrid Bianca undergoes hypersensitive response upon pathogen inoculation. Here, the transcriptome data (RNA-seq) previously obtained [17] were used to explore candidate pathways and genes that are differentially expressed by *P. viticola* during the interaction with two *V. vinifera* varieties characterized by susceptible (Pinot noir) and resistant (Mgaloblishvili) phenotypes and a resistant hybrid (Bianca).

Transcript abundance levels within an organism follow a log-normal distribution, meaning that a small percentage of all transcripts cover a large fraction of all the transcripts present in the cell at any given moment [21]. For this reason, transcriptomic studies require deeper sequencing in order to identify transcripts with lower than average expression levels. It should be noted that in dual-omics experiments, in which the transcriptomes of two organisms are screened at once, the problem is exacerbated. Thus, when mapping RNA reads on the *P. viticola* genome, only 5251 genes appear to be expressed, and most of them at very low levels. Nonetheless, the samples relative to the 3 dai time point contain enough reads to run differential expression analysis. Almost all of the *P. viticola* DEGs (99%) concerned the comparison of its transcriptome in Mgaloblishvili and Pinot noir versus Bianca, since the samples concerning *P. viticola* in Mgaloblishvili and Pinot noir are much more similar. The great similarity between the genes expressed by *P. viticola* in Mgaloblishvili and Pinot noir could be due to the fact that these are both *V. vinifera* varieties, while Bianca is characterized by the presence of 20% genes of American grapevine species [54]. Therefore, the comparison of *P. viticola* transcriptome on *V. vinifera* with a non-*vinifera* variety was mainly used to elucidate the genes involved in the plant–pathogen interaction. The biological functions associated with *P. viticola* DEGs are almost identical in Mgaloblishvili and Pinot noir and are mostly related to biosynthetic processes. Based on PvM_vs_PvB and PvP_vs_PvB contrasts, the *P. viticola* DE transcriptome on Mgaloblishvili differs from the one on Pinot noir mainly for the presence of GO terms related to response to stress, which could be associated with the action of the plant on the pathogen: at 3 dai *P. viticola* growth was, in fact, clearly hampered in Mgaloblishvili, while no alteration of the vegetative structures of the pathogen was observed in Pinot noir [17].

### 4.2. P. viticola Effectors

When analyzing the genes encoding effectors expressed by *P. viticola* in the comparison among grapevine accessions at 3 dai, it was possible to see that no CRN-encoding genes were differentially expressed by the pathogen, while eight transcripts with RXLR and YxSLK motif were up-regulated in both Mgaloblishvili and Pinot noir, and in Pinot noir compared to Bianca (PvP_vs_PvB). Among genes encoding apoplastic effectors, we found few NPP1 (three) and elicitin (seven) DEGs and several trypsin DEGs (17), associated with protein hydrolysis, up-regulated in Mgaloblishvili and/or Pinot noir. Finally, 76 DEGs encoding proteins with a signal peptide for secretion were found. Most of the differentially expressed genes encoding for effectors were up-regulated by the pathogen in both Mgaloblishvili and Pinot noir (57) or in PvP_vs_PvB (45) and only a few were up-regulated exclusively in Mgaloblishvili (seven) or Bianca (two).

Of the 15 apoplastic effectors and 38 secreted proteins encoded by genes with a known function up-regulated by *P. viticola* in both *V. vinifera* cultivars, many are proteins involved in protein hydrolysis and modification (glycosylation and folding), as previously found by other authors [21,27]. Another important category is formed by cell wall degrading enzymes (CWDEs) targeting cellulose, hemicellulose, and pectins [55], which constitute the primary defense of the plant cell, and β-1,3-glucans such as callose, which is produced by the plant in response to the pathogen as a physical barrier to penetration [56,57]. CWDEs are extracellular effectors that degrade a wide range of polysaccharides and glycoproteins not only during plant penetration but also to allow the release of nutrients [55].

Five genes encoding apoplastic effectors and two genes encoding secreted proteins were up-regulated by *P. viticola* exclusively during the interaction with Mgaloblishvili. These effectors are putatively involved in the hydrolysis of antifungal proteins produced by the host cell and cell wall degradation during colonization [58]. While β-(1,6)-glucanase activity has been detected during fungal pathogenesis, its role remains unclear as host plants lack its β-(1,6)-glucan substrate [59]. An explanation of the up-regulation of this gene could be related to the growth of *P. viticola* mycelium: β-glucanases play a role in cell wall growth and extension by partially hydrolyzing localized areas and enabling insertion of new cell wall material without disrupting its overall integrity [59]. Three days after inoculation on Mgaloblishvili, *P. viticola* indeed showed contorted, hyper-branched hyphae that could indicate a high cell wall synthesis [17]. These candidate effectors should be better investigated since they are specifically related to the immune response of the resistant *V. vinifera* cultivar.

Based on the comparative analysis of the *P. viticola* transcriptome, it was possible to identify four candidate cytoplasmic effectors with a specific role in pathogenesis on Pinot noir: three RXLR and an YxSLK. Of these effectors, only the YxSLK had a function assigned to it: it encodes a spermidine synthase, a protein catalyzing the biosynthesis of a polyamine involved in several fungal functions, such as dimorphism, spore germination and appressorium formation, and in virulence [60,61]. The transcripts encoding non-cytoplasmic effectors associated with susceptibility in Pinot noir showed functions analogous to those previously described and included also genes encoding enzymes involved in response to oxidative stress (monodehydroascorbate reductase (NADH), responsible for scavenging ROSs in plants and reducing phenoxyl radicals [62,63]), metabolism (6-phosphogluconolactonase, involved in the pentose phosphate pathway, a pathway highly represented in *P. infestans* hyphae [64]), and phosphate nutrition (acid phosphatase [65]). 

### 4.3. Networking Analysis Reveals A Putative Gene of Susceptibility in V. vinifera

Network analysis clustered genes in 10 coexpression modules (Figure 4). These modules showed enrichment of the most common plant–pathogen interaction pathways, such as plant hormone signal transduction and biosynthesis of secondary metabolites (monoterpenes and flavonoids) [66,67], confirming the results of the previous study [17].

The availability of a resistant *V. vinifera* accession provided us the opportunity to explore the genes associated with susceptibility within the species that, to the best of our knowledge, have not been discovered yet [68]. In plant–pathogen interaction, apart from R (resistance) genes, that can detect effectors and activate ETI, there are S (susceptibility) genes, which are required for successful pathogen infection and are considered essential for compatible plant–pathogen interaction [8]. S genes can be involved in early pathogen establishment, modulation of host defenses, and pathogen sustenance [68], and their disruption can confer disease resistance [8]. S genes are genes coding for effector targets that function as negative defense regulators or susceptibility factors [69]. One of the methods used for individuating S genes is based on gene expression studies, where inoculation with the pathogen results in the gene up-regulation during compatible interaction and transgenic overexpression of the gene leads to susceptibility [70]. It must be pointed out that S genes can show a constitutive expression and an increased expression upon pathogen inoculation [71]. To identify putative S genes in *V. vinifera*, the following hypothesis has been made: the putative S genes should have been down-regulated in Mgaloblishvili (resistant *V. vinifera*) inoculated samples in comparison with non-inoculated samples, and up-regulated in Pinot noir (susceptible *V. vinifera*) inoculated versus non-inoculated samples at 1 dai, the most informative time point in *P. viticola*-grapevine interaction [17]. Among the genes examined, only a gene encoding a LOB domain-containing (LBD) protein (XM_010660137.1) showed these characteristics. In *Arabidopsis*, the root-specific LBD transcription factor AtLBD20 acts as a repressor of a subset of jasmonate-mediated defense mechanisms during infection of roots with *Fusarium oxysporum* Schltdl., and the knockout of this gene resulted in increased resistance to *F. oxysporum* [72]. The down-regulation of this gene in Mgaloblishvili and its up-regulation in Pinot noir make it a candidate S gene associated with a negative regulation of immunity signals [8,69]. Further studies are needed to confirm this hypothesis.

### 4.4. De novo Transcriptome Assembly Reveals New Grapevine Traits Related to P. viticola Response

*V. vinifera* is one of the most highly heterozygous species and has high genetic diversity [73] such that the commonly used [17,74,75,76,77] reference genome PN40024 12X v2 [78] is not comprehensive of the entire complexity of modern cultivars. For this reason, de novo transcriptome assembly of Mgaloblishvili, Pinot noir, and Bianca was performed to characterize the different responses to *P. viticola* infection. The largest number of unique transcripts was detected in Bianca samples, as expected due to its non-*vinifera* background [54], while the lowest was in Pinot noir (Table S7). 

The GO analysis showed that most of the unique transcripts of Mgaloblishvili inoculated samples were involved in the processes of “phosphorylation”, “integral component of membrane”, and “nucleic acid binding” (Figure S3), that can be related to signaling, recognition, and transcriptional regulation in the plant–pathogen interaction [79,80,81,82,83].

In Pinot noir, most of the unique transcripts of inoculated samples were involved in “cellular protein modification” and “protein binding” (Figure S4) with a significant role in the plant–pathogen interaction [83]. 

Unlike Mgaloblishvili, Bianca showed a higher enrichment of GO categories related to the “oxidation–reduction biological process” and “hydrolase activity” (Figure S5) due to the ability of Bianca to trigger a localized HR and secrete hydrolytic enzymes (such as PR proteins, glucanases, chitinases, and proteases) soon after the onset of infection to counteract the pathogen invasion [56,84,85,86]. 

### 4.5. Transcriptomic Changes Occurring In Grapevine During The First Stages of P. viticola Infection

As already found in similar studies [12,87], 1 dai was the most informative time point in our experimental conditions (Table 3; Tables S8-S10) [17]. This result is strictly related to the infection process, when the haustorium and the plant cell membrane get in contact for the first time [17,56,88]. The number of DEGs was larger for Bianca samples, followed by Mgaloblishvili and Pinot noir samples. This trend is correlated to the genotype resistance level against the pathogen. Bianca is a resistant variety showing HR [56,84,85] and promptly (at 1 dai) activates the molecular mechanisms preventing mycelial growth [89]. Mgaloblishvili showed a down regulation of *P. viticola* growth and sporulation associated with PAMP, DAMP, and effector recognition [17]. Whereas Pinot noir is a susceptible variety, not able to counteract pathogen growth [17,87].

### 4.6. Unique DEGs of Mgaloblishvili in Response to P. viticola Infection 

To elucidate the Mgaloblishvili response to *P. viticola*, unique DEGs having a LFC value above the threshold were discussed (Table S8). Among up-regulated DEGs, five genes, encoding two receptor-like kinase (RLKs) proteins, two 18.3 kDa heat shock proteins, and valencene synthase, were strictly linked to the plant–pathogen interaction mechanism [17]. RLKs (receptor-like kinases) appear to play a central role in the plant–pathogen interaction, signaling during pathogen recognition, and activation of the plant defense mechanisms [90]. Synthesis of small heat shock proteins (sHSPs), with a molecular mass of 15–42 kD, can be induced by heat shock and various abiotic or biotic stresses [91,92] and are involved in the initial stages of fungal recognition in HR-independent defense mechanism [93]. Valencene synthase (already identified in previous study [17]) catalyzes the conversion of farnesyl diphosphate to valencene, a sesquiterpene with antimicrobial activity [94,95,96]. 

Among the Mgaloblishvili down-regulated DEGs, only one transcript appeared to be strictly linked to the plant–pathogen interaction mechanism, and not already identified in the previous study [17]. It encodes a probable xyloglucan endotransglucosylase/hydrolase (XTH) protein 32. XTHs are a class of cell wall-modifying proteins involved in cell growth, having the ability to loosen cell walls [97,98]. The overexpression of these genes makes the membrane more permeable [99]. Membrane fluidity has a role in the *P. viticola*–grapevine interaction, where a more rigid membrane is associated with resistance to the pathogen [100]. Therefore, the down-regulation of a XTH gene could likely be a part of the resistance mechanism of Mgaloblishvili by increasing the compactness of cells and membrane permeability.

## 5. Conclusions 

In conclusion, we identified the dynamics of gene and pathway regulation in *P. viticola* and susceptible/resistant grapevines during the battle for infection. On the one hand, during the interaction with Mgaloblishvili, *P. viticola* specifically up-regulated genes encoding apoplastic effectors and putative proteases that were not differentially expressed during the interaction with the susceptible (Pinot noir) and resistant (Bianca) grapevine cultivars. On the other hand, Mgaloblishvili confirmed its unique response to *P. viticola* infection based on the up-regulation of resistance genes involved in signaling, induction of basal immune response, and metabolism of terpenes, as previously described [17]. Moreover, the de novo assembly of the grapevine accessions allowed us to identify new genes that can be at the basis of *V. vinifera* resistance to *P. viticola*.

Apart from the detection of genes putatively involved in resistance, this study also highlighted the presence of a possible susceptibility gene, a transcription factor that was down-regulated in the resistant *V. vinifera* cv. Mgaloblishvili, up-regulated in the susceptible *V. vinifera* cv. Pinot noir, and not differentially expressed in the resistant hybrid Bianca. New perspectives in genetic improvement of grapevine for resistance to *P. viticola* are needed, and in this regard the exploitation of susceptibility genes could be remarkable. Recently, disrupting of S genes to interfere with the host–pathogen compatibility and obtaining disease resistance has become of great interest, due to the accomplishment of this task in a transgene-free way via new genome editing tools [101].

The results obtained in the present study contribute to elucidate the mechanisms of interaction between *P. viticola* and *V. vinifera* during infection. Further analyses are needed to investigate the role of the candidate effectors in pathogenicity through functional analysis and to confirm the role of the putative resistance/susceptibility genes of *V. vinifera*. 

## Figures and Tables

**Table 1:** Number (Nr.) and percentage (%) of differentially expressed genes (DEGs) by *Plasmopara viticola* (Pv) when inoculated on Mgaloblishvili (M) in comparison with Pinot noir (P) (PvM_*vs*_PvP), Mgaloblishvili in comparison with Bianca (B) (PvM_*vs*_PvB), and Pinot noir in comparison with Bianca (PvP_*vs*_PvB).

DEGs	Contrast						Total	
	PvM_*vs*_PvP		PvM_*vs*_PvB		PvP_*vs*_PvB			
	Nr.	%	Nr.	%	Nr.	%	Nr.	%
*Up*	1	0.065	524	34.5	939	61.82	1464	96.39
*Down*	1	0.065	9	0.59	45	2.96	55	3.62
Total	2	0.13	533	35.09	984	64.78	1519	100

**Table 2:** Number of de novo transcripts assembled by Trinity for each condition and genotype analyzed. The run accession numbers associated with the respective sample type are reported. Supertranscripts indicate the number of transcripts after collapsing unique and common sequence regions among splicing isoforms.

Library Run accession (Study PRJEB24540; European Nucleotide Archive)	Library Type (Cultivar Name, Treatment, dai Number)	Number of Reads Passing Trimmomatic Filter for Trinity de novo Assembly	Number of Transcripts Assembled by Trinity	Number of Supertranscripts Assembled by Trinity
ERR2274751, ERR2274752, ERR2274753	Mgaloblishvili, non-inoculated, 0 dai	77,329,414	48,263	33,701
ERR2274754, ERR2274755, ERR2274756	Mgaloblishvili, non-inoculated, 1 dai	77,053,567	43,102	31,513
ERR2274757, ERR2274758, ERR2274759	Mgaloblishvili, non-inoculated, 2 dai	79,505,005	47,912	33,969
ERR2274760, ERR2274761, ERR2274762	Mgaloblishvili, non-inoculated, 3 dai	59,610,375	44,152	32,075
ERR2276774, ERR2276775, ERR2276776	Mgaloblishvili, inoculated, 1 dai	80,263,242	50,930	34,701
ERR2276777, ERR2276778, ERR2276779	Mgaloblishvili, inoculated, 2 dai	74,298,111	51,109	35,146
ERR2276780, ERR2276781, ERR2276782	Mgaloblishvili, inoculated, 3 dai	86,910,171	43,151	31,905
ERR2262368, ERR2262369, ERR2262534	Pinot noir, non-inoculated, 0 dai	77,508,661	38,749	28,818
ERR2265572, ERR2265573, ERR2265574	Pinot noir, non-inoculated, 1 dai	71,622,301	44,433	32,403
ERR2265575, ERR2265576, ERR2265577	Pinot noir, non-inoculated, 2 dai	71,910,056	45,555	32,779
ERR2265578, ERR2265579, ERR2265580	Pinot noir, non-inoculated, 3 dai	72,205,904	47,860	35,603
ERR2262591, ERR2262592, ERR2262593	Pinot noir, inoculated, 1 dai	72,980,600	40,313	30,198
ERR2264780, ERR2264781, ERR2264782	Pinot noir, inoculated, 2 dai	72,188,628	47,313	34,683
ERR2265538, ERR2265539, ERR2265540	Pinot noir, inoculated, 3 dai	81,804,077	45,398	34,396
ERR2271536, ERR2271537, ERR2271538	Bianca, non-inoculated, 0 dai	83,267,610	55,833	35,120
ERR2271539, ERR2271540, ERR2271541	Bianca, non-inoculated, 1 dai	73,434,003	40,946	30,225
ERR2271542, ERR2271543, ERR2271544	Bianca, non-inoculated, 2 dai	81,386,614	54,370	34,995
ERR2271545, ERR2271546, ERR2271547	Bianca, non-inoculated, 3 dai	79,917,013	59,852	37,236
ERR2276787, ERR2276788, ERR2276789	Bianca, inoculated, 1 dai	78,491,558	57,665	36,349
ERR2276790, ERR2276791, ERR2276792	Bianca, inoculated, 2 dai	79,960,138	56,124	35,554
ERR2276793, ERR2276794, ERR2276795	Bianca, inoculated, 3 dai	84,125,204	60,459	37,891

**Table 3:** Overview of differentially expressed genes (Nr. and %) detected in Mgaloblishvili, Pinot noir, and Bianca leaves inoculated with *Plasmopara viticola*, at three different time points (1, 2, and 3 dai), based on the de novo transcriptome. dai: day after inoculation.

Cultivar		Differentially Expressed Genes							
		1 dai			2 dai			3 dai	
		Nr.	%		Nr.	%		Nr.	%
Mgaloblishvili									
*Up*		2592	4.20		162	0.26		73	0.12
*Down*		3976	6.50		40	0.06		87	0.14
Pinot noir									
*Up*		1349	2.50		769	1.30		4905	8.50
*Down*		1349	2.50		145	0.25		4551	7.80
Bianca									
*Up*		6564	8.90		256	0.34		5789	7.50
*Down*		9884	13.0		228	0.30		5327	6.90

## Supplementary Materials

The following are available online at https://www.mdpi.com/2073-4425/11/3/261/s1, Table S1. List of transcripts differentially expressed by *P. viticola* upon inoculation of Mgaloblishvili in comparison with Pinot noir (PvM_*vs*_PvP), Mgaloblishvili in comparison with Bianca (PvM_*vs*_PvB), and Pinot noir in comparison with Bianca (PvP_*vs*_PvB), three days after inoculation, Table S2. List of the GO terms related to up- and down-regulated differentially expressed genes expressed by *P. viticola* upon inoculation of Mgaloblishvili in comparison with Bianca (PvM_*vs*_PvB) and Pinot noir in comparison with Bianca (PvP_*vs*_PvB), three days after inoculation, Table S3. List of the differentially expressed genes encoding cytoplasmic and apoplastic effectors and proteins with signal peptide for secretion up- and down-regulated by *P. viticola* upon inoculation of Mgaloblishvili in comparison with Pinot noir (PvM_*vs*_PvP), Mgaloblishvili in comparison with Bianca (PvM_*vs*_PvB), and Pinot noir in comparison with Bianca (PvP_*vs*_PvB), three days after inoculation, Table S4. Enriched pathway terms in modules detected via the network analysis represented in Figure 4, Table S5. List of transcription factors (TFs) detected in each module of network analysis represented in Figure 4, Table S6. Transcription factors (TFs) enrichment of module genes detected via network analysis represented in Figure 4, Table S7. List of transcripts of three grapevine cultivars (Mgaloblishvili, Pinot noir, and Bianca), inoculated and non-inoculated with *P. viticola* used for GO analysis, Table S8. List of unique differentially expressed genes identified in Mgaloblishvili inoculated with *P. viticola* at 1, 2, and 3 days after inoculation. dai = day after inoculation, Table S9. List of unique differentially expressed genes identified in Pinot noir inoculated with *P. viticola* at 1, 2, and 3 days after inoculation. dai = day after inoculation, Table S10. List of unique differentially expressed genes identified in Bianca inoculated with *P. viticola* at 1, 2, and 3 days after inoculation. dai = day after inoculation, Figure S1. Heatmap of normalized expression values of genes of yellow module detected by network analysis (Figure 4) for Mgaloblishvili, Pinot noir, and Bianca inoculated and non-inoculated with *P. viticola* at 1, 2, and 3 days after inoculation, Figure S2. Heatmap of normalized expression values of genes of blue module detected by network analysis (Figure 4) for Mgaloblishvili, Pinot noir, and Bianca inoculated and non-inoculated with *P. viticola* at 1, 2, and 3 days after inoculation, Figure S3. Overview of the most enriched GO categories in inoculated vs. non-inoculated samples of Mgaloblishvili. A: Biological process GO categories; B: cellular component GO categories; C: molecular function GO categories, Figure S4. Overview of the most enriched GO categories in inoculated vs. non-inoculated samples of Pinot noir. A: Biological process GO categories; B: cellular component GO categories; C: molecular function GO categories, Figure S5. Overview of the most enriched GO categories in inoculated vs. non-inoculated samples of Bianca. A: Biological process GO categories; B: cellular component GO categories; C: molecular function GO categories, Figure S6. Comparison between differentially expressed genes (DEGs) of Mgaloblishvili samples inoculated with *P. viticola*, at 1, 2, and 3 days after inoculation. M = Mgaloblishvili; i = inoculated; dai = day after inoculation, Figure S7. Comparison between differentially expressed genes (DEGs) of Pinot noir samples inoculated with *P. viticola*, at 1, 2, and 3 days after inoculation. P = Pinot noir; i = inoculated; dai = day after inoculation, Figure S8. Comparison between differentially expressed genes (DEGs) of Bianca samples inoculated with *P. viticola*, at 1, 2, and 3 days after inoculation. B = Bianca; i = inoculated; dai=day after inoculation.

## References

[1] Jacobsen B.J. (1997). Role of plant pathology in Integrated Pest Management. Annu. Rev. Phytopathol..

[2] Molitor D., Biewers B., Junglen M., Schultz M., Clement P., Permesang G., Regnery D., Porten M., Herzog K., Hoffmann L. (2018). Multi-annual comparisons demonstrate differences in the bunch rot susceptibility of nine *Vitis vinifera* L. ’Riesling’ clones. Vitis.

[3] Rotolo C., De Miccolis Angelini R.M., Dongiovanni C., Pollastro S., Fumarola G., Di Carolo M., Perrelli D., Natale P., Faretra F. (2018). Use of biocontrol agents and botanicals in integrated management of *Botrytis cinerea* in table grape vineyards. Pest Manag. Sci..

[4] Passera A., Compant S., Casati P., Maturo M.G., Battelli G., Quaglino F., Antonielli L., Salerno D., Brasca M., Toffolatti S.L. (2019). Not just a pathogen? Description of a plant-beneficial *Pseudomonas syringae* strain. Front. Microbiol..

[5] Toffolatti S.L., Russo G., Campia P., Bianco P.A., Borsa P., Coatti M., Torriani S.F., Sierotzki H. (2018). A time-course investigation of resistance to the carboxylic acid amide mandipropamid in field populations of *Plasmopara viticola* treated with anti-resistance strategies. Pest Manag. Sci..

[6] Boyd L.A., Ridout C., O’Sullivan D.M., Leach J.E., Leung H. (2013). Plant–pathogen interactions: Disease resistance in modern agriculture. Trends Genet..

[7] REX Consortium (2016). Combining selective pressures to enhance the durability of disease resistance genes. Front. Plant Sci..

[8] Zaidi S.S., Mukhtar M.S., Mansoor S. (2018). Genome editing: Targeting susceptibility genes for plant disease resistance. Trends Biotechnol..

[9] McGovern P.E., Jalabadze M., Batiuk S., Callahan M.P., Smith K.E., Hall G.R., Kvavadze E., Maghradze D., Rusishvili N., Bouby L. (2017). Early Neolithic wine of Georgia in the South Caucasus. Proc. Natl. Acad. Sci. USA.

[10] Emmett R.W., Wicks T.J., Magarey P.A., Kumar J., Chaube H.S., Singh U.S., Mukhopadhyay A.N. (1992). Downy mildew of grapes. Plant Diseases of International Importance—Diseases of Fruit Crops.

[11] Gessler C., Pertot I., Perazzolli M. (2011). *Plasmopara viticola*: A review of knowledge on downy mildew of grapevine and effective disease management. Phytopathol. Mediterr..

[12] Buonassisi D., Colombo M., Migliaro D., Dolzani C., Peressotti E., Mizzotti C., Velasco R., Masiero S., Perazzolli M., Vezzulli S. (2017). Breeding for grapevine downy mildew resistance: A review of “omics” approaches. Euphytica.

[13] Merdinoglu D., Schneider C., Prado E., Wiedemann-Merdinoglu S., Mestre P. (2018). Breeding for durable resistance to downy and powdery mildew in grapevine. OENO One.

[14] Boubals D. (1959). Contribution a l’etude des causes de la resistance des Vitacees au mildiou de la vigne (*Plasmopara viticola* (B. *et* C.) Berl. *et* de T.) et leur mode de transmission hereditaire. Ann. Amélior. Plant..

[15] Pedneault K., Provost C. (2016). Fungus resistant grape varieties as a suitable alternative for organic wine production: Benefits, limits, and challenges. Sci. Hortic..

[16] Toffolatti S.L., Maddalena G., Salomoni D., Maghradze D., Bianco P.A., Failla O. (2016). Evidence of resistance to the downy mildew agent *Plasmopara viticola* in the Georgian *Vitis vinifera* germplasm. Vitis.

[17] Toffolatti S.L., De Lorenzis G., Costa A., Maddalena G., Passera A., Bonza M.C., Pindo M., Stefani E., Cestaro A., Casati P. (2018). Unique resistance traits against downy mildew from the center of origin of grapevine (*Vitis vinifera*). Sci. Rep..

[18] Rovenich H., Boshoven J.C., Thomma B.P.H.J. (2014). Filamentous pathogen effector functions: Of pathogens, hosts and microbiomes. Curr. Opin. Plant Biol..

[19] Kamoun S. (2007). Groovy times: Filamentous pathogen effectors revealed. Curr. Opin. Plant Biol..

[20] Franceschetti M., Maqbool A., Jiménez-Dalmaroni M.J., Pennington H.G., Kamoun S., Banfield M.J. (2017). Effectors of filamentous plant pathogens: Commonalities amid diversity. Microbiol. Mol. Biol. Rev..

[21] Brilli M., Asquini E., Moser M., Bianchedi P.L., Perazzolli M., Si-Ammour A. (2018). A multi-omics study of the grapevine-downy mildew (*Plasmopara viticola*) pathosystem unveils a complex protein coding and noncoding-based arms race during infection. Sci. Rep..

[22] Mestre P., Piron M.C., Merdinoglou D. (2012). Identification of effector genes from the phytopathogenic Oomycete *Plasmopara viticola* through the analysis of gene expression in germinated zoospores. Fungal Biol..

[23] Mestre P., Carrere S., Gouzy J., Piron M.-C., Tourvieille de Labrouhee D., Vincourt P., Delmotte F., Godiard L. (2016). Comparative analysis of expressed CRN and RXLR effectors from two *Plasmopara* species causing grapevine and sunflower downy mildew. Plant Pathol..

[24] Xiang J., Li X., Wu J., Yin L., Zhang Y., Lu J. (2016). Studying the mechanism of *Plasmopara viticola* RxLR effectors on suppressing plant immunity. Front. Microbiol..

[25] Xiang J., Li X., Yin L., Liu Y., Zhang Y., Qu J., Lu J. (2017). A candidate RxLR effector from *Plasmopara viticola* can elicit immune responses in *Nicotiana benthamiana*. BMC Plant Biol..

[26] Yin L., Li X., Xiang J., Qu J., Zhang Y., Dry I.B., Lu J. (2015). Characterization of the secretome of *Plasmopara viticola* by *de novo* transcriptome analysis. Physiol. Mol. Plant Pathol..

[27] Yin L., An Y., Qu J., Li X., Zhang Y., Dry I., Wu H., Lu J. (2017). Genome sequence of *Plasmopara viticola* and insight into the pathogenic mechanism. Sci. Rep..

[28] Stam R., Jupe J., Howden A.J.M., Morris J.A., Boevink P.C., Hedley P.E., Huitema E. (2013). Identification and characterisation of CRN effectors in *Phytophthora capsici* shows modularity and functional diversity. PLoS ONE.

[29] Amaro T.M.M.M., Thilliez G.J.A., Motion G.B., Huitema E. (2017). A perspective on CRN proteins in the genomics age: Evolution, classification, delivery and function revisited. Front. Plant Sci..

[30] Lévesque C.A., Brouwer H., Cano L., Hamilton J.P., Holt C., Huitema E., Raffaele S., Robideau G.P., Thines M., Win J. (2010). Genome sequence of the necrotrophic plant pathogen *Pythium ultimum* reveals original pathogenicity mechanisms and effector repertoire. Genome Biol..

[31] McGowan J., Fitzpatrick D.A. (2017). Genomic, network, and phylogenetic analysis of the oomycete effector arsenal. mSphere.

[32] Leesutthiphonchai W., Vu A.L., Ah-Fong A.M.V., Judelson H.S. (2018). How does *Phytophthora infestans* evade control efforts? Modern insight into the late blight disease. Phytopathology.

[33] Petre B., Kamoun S. (2014). How do filamentous pathogens deliver effector proteins into plant cells?. PLoS Biol..

[34] Wang S., Boevink P.C., Welsh L., Zhang R., Whisson S.C., Birch P.R.J. (2017). Delivery of cytoplasmic and apoplastic effectors from *Phytophthora infestans* haustoria by distinct secretion pathways. New Phytol..

[35] Kamoun S. (2006). A catalogue of the effector secretome of plant pathogenic Oomycetes. Annu. Rev. Phytopathol..

[36] Kanneganti T.-D., Huitema E., Cakir C., Kamoun S. (2006). Synergistic interactions of the plant cell death pathways induced by *Phytophthora infestans* Nep1-Like Protein PiNPP1.1 and INF1 Elicitin. MPMI.

[37] Dangl J.L., Horvath D.M., Staskawicz B.J. (2013). Pivoting the plant immune system from dissection to deployment. Science.

[38] Giraldo M.C., Valent B. (2013). Filamentous plant pathogen effectors in action. Nat. Rev. Microbiol..

[39] Miller R.N.G., Alves G.S.C., Van Sluys M.-A. (2017). Plant immunity: Unravelling the complexity of plant responses to biotic stresses. Ann. Bot..

[40] *Plasmopara viticola* Multi Omics Dissection. https://www.researchgate.net/project/Plasmopara-viticola-multi-omics-dissection.

[41] Tian T., Liu Y., Yan H., You Q., Yi X., Du Z., Xu W., Su Z. (2017). agriGO v2.0: A GO analysis toolkit for the agricultural community, 2017 update. Nucleic Acids Res..

[42] Supek F., Bošnjak M., Škunca N., Šmuc T. (2011). REVIGO summarizes and visualizes long lists of Gene Ontology terms. PLoS ONE.

[43] Bardou P., Mariette J., Escudié F., Djemiel C., Klopp C. (2014). jvenn: An interactive Venn diagram viewer. BMC Bioinform..

[44] Warnes G.R., Bolker B., Lumley T. (2009). gplots: Various R Programming Tools for Plotting Data. http://cran.r-project.org/web/packages/gplots/index.html.

[45] Love M.I., Huber W., Anders S. (2014). Moderated estimation of fold change and dispersion for RNA-Seq data with DESeq2. Genome Biol..

[46] Langfelder P., Horvath S. (2008). WGCNA: An R package for weighted correlation network analysis. BMC Bioinform..

[47] Ravasz E., Somera A.L., Mongru D.A., Oltvai Z.N., Barabasi A.L. (2002). Hierarchical organization of modularity in metabolic networks. Science.

[48] Langfelder P. (2013). Signed vs. Unsigned Topological Overlap Matrix. Technical Report. https://horvath.genetics.ucla.edu/html/CoexpressionNetwork/Rpackages/WGCNA/TechnicalReports/signedTOM.pdf.

[49] Grabherr M.G., Haas B.J., Yassour M., Levin J.Z., Thompson D.A., Amit I., Adiconis X., Fan L., Raychowdhury R., Zeng Q. (2011). Full-length transcriptome assembly from RNA-Seq data without a reference genome. Nat. Biotechnol..

[50] The Kore cluster. http://sit.fbk.eu/hpc/kore.

[51] Fu L., Niu B., Zhu Z., Wu S., Li W. (2012). CD-HIT: Accelerated for clustering the next-generation sequencing data. Bioinformatics.

[52] Conesa A., Götz S., García-Gómez J.M., Terol J., Talón M., Robles M. (2005). Blast2GO: A universal tool for annotation, visualization and analysis in functional genomics research. Bioinformatics.

[53] Langmead B., Salzberg S. (2012). Fast gapped-read alignment with Bowtie 2. Nat. Methods.

[54] Di Gaspero G., Cipriani G. (2002). Resistance gene analogs are candidate markers for disease-resistance genes in grape (*Vitis* spp.). Theor. Appl. Genet..

[55] Blackman L.M., Cullerne D.P., Hardham A.R. (2014). Bioinformatic characterisation of genes encoding cell wall degrading enzymes in the *Phytophthora parasitica* genome. BMC Genom..

[56] Toffolatti S.L., Venturini G., Maffi D., Vercesi A. (2012). Phenotypic and histochemical traits of the interaction between *Plasmopara viticola* and resistant or susceptible grapevine varieties. BMC Plant Biol..

[57] Gauthier A., Trouvelot S., Kelloniemi J., Frettinger P., Wendehenne D., Daire X., Joubert J.M., Ferrarini A., Delledonne M., Flors V. (2014). The sulfated laminarin triggers a stress transcriptome before priming the SA- and ROS-dependent defenses during grapevine’s induced resistance against *Plasmopara viticola*. PLoS ONE.

[58] Legay G., Marouf E., Berger D., Neuhaus J.-M., Mauch-Mani B., Slaughter A. (2001). Identification of genes expressed during the compatible interaction of grapevine with *Plasmopara viticola* through suppression subtractive hybridization (SSH). Eur. J. Plant Pathol..

[59] Martin K., McDougall B.M., McIlroy S., Jayus Chen J., Seviour R.J. (2007). Biochemistry and molecular biology of exocellular fungal β-(1,3)- and β-(1,6)-glucanases. FEMS Microbiol. Rev..

[60] Valdés-Santiago L., Cervantes-Chávez J.A., León-Ramírez C.G., Ruiz-Herrera J. (2012). Polyamine metabolism in fungi with emphasis on phytopathogenic species. J. Amino Acids.

[61] Majumdar R., Lebar M., Mackm B., Minocha R., Minocha S., Carter-Wientjes C., Sickler C., Rajasekaran K., Cary J.W. (2018). The *Aspergillus flavus* Spermidine Synthase (spds) Gene, is required for normal development, aflatoxin production, and pathogenesis during infection of maize kernels. Front. Plant Sci..

[62] Sakihama Y., Mano J., Sano S., Asada K., Yamasaki H. (2000). Reduction of Phenoxyl Radicals mediated by Monodehydroascorbate Reductase. Biochem. Biophys. Res. Commun..

[63] Wojtaszek P. (1997). Oxidative burst: An early plant response to pathogen infection. Biochem. J..

[64] Ah-Fong A.M.V., Su Kim K., Judelson H.S. (2017). RNA-seq of life stages of the oomycete *Phytophthora infestans* reveals dynamic changes in metabolic, signal transduction, and pathogenesis genes and a major role for calcium signaling in development. BMC Genom..

[65] Aliaga G.R., Ellzey J.T. (1984). Ultrastructural localization of acid phosphatase and alkaline phosphatase within oogonia of *Achlya recurve*. Mycologia.

[66] Lapin D., Van den Ackerveken G. (2014). Susceptibility to plant disease: More than a failure of host immunity. Trends Plant Sci..

[67] Chitarrini G., Soini E., Riccadonna S., Franceschi P., Zulini L., Masuero D., Vecchione A., Stefanini M., Di Gaspero G., Mattivi F. (2017). Identification of biomarkers for defense response to *Plasmopara viticola* in a resistant grape variety. Front. Plant Sci..

[68] Fawke S., Doumane M., Schornack S. (2015). Oomycete interactions with plants: Infection strategies and resistance principles. Microbiol. Mol. Biol. Rev..

[69] Pavan S., Jacobsen E., Visser R.G.F., Bai Y. (2010). Loss of susceptibility as a novel breeding strategy for durable and broad-spectrum resistance. Mol. Breed..

[70] Van Schie C.C.N., Takken F.L.W. (2014). Susceptibility Genes 101: How to Be a Good Host. Annu. Rev. Phytopathol..

[71] Weis C., Pfeilmeier S., Glawischnig E., Isono E., Pachl F., Hahne H., Kuster B., Eichmann R., Hückelhoven R. (2013). Co-immunoprecipitation-based identification of putative BAX INHIBITOR-1-interacting proteins involved in cell death regulation and plant-powdery mildew interactions. Mol. Plant Pathol..

[72] Thatcher L.F., Powell J.J., Aitken E.A., Kazan K., Manners J.M. (2012). The lateral organ boundaries domain transcription factor LBD20 functions in *Fusarium* wilt susceptibility and jasmonate signaling in *Arabidopsis*. Plant Physiol..

[73] This P., Lacombe T., Thomas M.R. (2006). Historical origins and genetic diversity of wine grapes. Trends Genet..

[74] Corso M., Vannozzi A., Maza E., Vitulo N., Meggio F., Pitacco A., Telatin A., D’Angelo M., Feltrin E., Negri A.S. (2015). Comprehensive transcript profiling of two grapevine rootstock genotypes contrasting in drought susceptibility links the phenylpropanoid pathway to enhanced tolerance. J. Exp. Bot..

[75] Vannozzi A., Donnini S., Vigani G., Corso M., Valle G., Vitulo N., Bonghi C., Zocchi G., Lucchin M. (2016). Transcriptional characterization of a widely-used grapevine rootstock genotype under different iron-limited conditions. Front. Plant Sci..

[76] Weng K., Li Z.-Q., Liu R.-Q., Wang L., Wang Y.-J., Xu Y. (2014). Transcriptome of *Erysiphe necator*-infected *Vitis pseudoreticulata* leaves provides insight into grapevine resistance to powdery mildew. Hortic. Res..

[77] Yang Y., Mao L., Jittayasothorn Y., Kang Y., Jiao C., Fei Z., Zhong G.-Y. (2015). Messenger RNA exchange between scions and rootstocks in grafted grapevines. BMC Plant Biol..

[78] Vitulo N., Forcato C., Carpinelli E.C., Telatin A., Campagna D., D’Angelo M., Zimbello R., Corso M., Vannozzi A., Bonghi C. (2014). A deep survey of alternative splicing in grape reveals changes in the splicing machinery related to tissue, stress condition and genotype. BMC Plant Biol..

[79] Ardito F., Giuliani M., Perrone D., Troiano G., Lo Muzio L. (2017). The crucial role of protein phosphorylation in cell signaling and its use as targeted therapy (Review). Int. J. Mol. Med..

[80] Xing T., Ouellet T., Miki B.L. (2002). Towards genomic and proteomic studies of protein phosphorylation in plant-pathogen interactions. Trends Plant Sci..

[81] Yang Y., Shah J., Klessig D.F. (1997). Signal perception and transduction in plant defense responses. Genes Dev..

[82] Yangnan G., Zavaliev R., Dong X. (2017). Membrane trafficking in plant immunity. Mol. Plant..

[83] Dangl J.L., Jones J.D.G. (2001). Plant pathogens and integrated defence responses to infection. Nature.

[84] Bellin D., Peressotti E., Merdinoglu D., Wiedemann-Merdinoglu S., Adam-Blondon A.-F., Cipriani G., Morgante M., Testolin R., Di Gaspero G. (2009). Resistance to *Plasmopara viticola* in grapevine “Bianca” is controlled by a major dominant gene causing localised necrosis at the infection site. Theor. Appl. Genet..

[85] Di Gaspero G., Copetti D., Coleman C., Castellarin S.D., Eibach R., Kozma P., Lacombe T., Gambetta G., Zvyagin A., Cindrić P. (2012). Selective sweep at the Rpv3 locus during grapevine breeding for downy mildew resistance. Theor. Appl. Genet..

[86] Fröbel S., Dudenhöffer J., Töpfer R., Zyprian E. (2019). Transcriptome analysis of early downy mildew (*Plasmopara viticola*) defense in grapevines carrying the Asian resistance locus *Rpv10*. Euphytica.

[87] Polesani M., Bortesi L., Ferrarini A., Zamboni A., Fasoli M., Zadra C., Lovato A., Pezzotti M., Delledonne M., Polverari A. (2010). General and species-specific transcriptional responses to downy mildew infection in a susceptible (*Vitis vinifera*) and a resistant (*V. riparia*) grapevine species. BMC Genom..

[88] Jürges G., Kassemeyer H.H., Durrenberger M., Duggelin M., Nick P. (2009). The mode of interaction between *Vitis* and *Plasmopara viticola* Berk. & Curt. Ex de Bary depends on the host species. Plant Biol..

[89] Peressotti E., Wiedemann-Merdinoglu S., Delmotte F., Bellin D., Di Gaspero G., Testolin R., Merdinoglu D., Mestre P. (2010). Breakdown of resistance to grapevine downy mildew upon limited deployment of a resistant variety. BMC Plant Biol..

[90] Afzal A.J., Wood A.J., Lightfoot D.A. (2008). Plant receptor-like serine threonine kinases: Roles in signaling and plant defense. Mol. Plant Microbe Interact..

[91] Whitham S.A., Yang C., Goodin M.M. (2006). Global impact: Elucidating plant responses to viral infection. Mol. Plant Microbe Interact..

[92] Maimbo M., Ohnishi K., Hikichi Y., Yoshioka H., Kiba A. (2007). Induction of a small heat shock protein and its functional roles in *Nicotiana* plants in the defense response against *Ralstonia solanacearum*. Plant Physiol..

[93] Fang X., Chen W., Xin Y., Zhang H., Yan C., Yu H., Liu H., Xiao W., Wang S., Zheng G. (2012). Proteomic analysis of strawberry leaves infected with *Colletotrichum fragariae*. J. Proteom..

[94] Manter D.K., Karchesy J.J., Kelsey R.G. (2006). The sporicidal activity of yellow-cedar heartwood, essential oil and wood constituents toward *Phytophthora ramorum* in culture. Forest Pathol..

[95] Sharon-Asa L., Shalit M., Frydman A., Bar E., Holland D., Or E., Lavi U., Lewinsohn E., Eyal Y. (2003). Citrus fruit flavor and aroma biosynthesis: Isolation, functional characterization, and developmental regulation of Cstps1, a key gene in the production of the sesquiterpene aroma compound valencene. Plant J..

[96] Lücker J., Bowen P., Bohlmann J. (2004). *Vitis vinifera* terpenoid cyclases: Functional identification of two sesquiterpene synthase cDNAs encoding (+)-valencene synthase and (−)-germacrene D synthase and expression of mono- and sesquiterpene synthases in grapevine flowers and berries. Phytochemistry.

[97] Nishitani K., Vissenberg K., Verbelen J.-P., Vissenberg K. (2007). Roles of the XTH protein family in the expanding cell. The Expanding Cell - Plant Cell Monographs.

[98] Maris A., Suslov D., Fry S.C., Verbelen J.-P., Vissenberg K. (2009). Enzymic characterization of two recombinant xyloglucan endotransglucosylase/hydrolase (XTH) proteins of Arabidopsis and their effect on root growth and cell wall extension. J. Exp. Bot..

[99] Han Y., Ban Q., Li H., Hou Y., Jin M., Han S., Rao J. (2016). DkXTH8, a novel xyloglucan endotransglucosylase/hydrolase in persimmon, alters cell wall structure and promotes leaf senescence and fruit postharvest softening. Sci. Rep..

[100] Laureano G., Figueiredo J., Cavaco A.R., Duarte B., Caçador I., Malhó R., Sousa Silva M., Matos A.R., Figueiredo A. (2018). The interplay between membrane lipids and phospholipase A family members in grapevine resistance against *Plasmopara viticola*. Sci. Rep..

[101] Pessina S., Lenzi L., Perazzolli M., Campa M., Dalla Costa L., Urso S., Valè G., Salamini F., Velasco R., Malnoy M. (2016). Knockdown of MLO genes reduces susceptibility to powdery mildew in grapevine. Hortic. Res..

