# Supplementary material for: Novel Aspects on The Interaction Between Grapevine and Plasmopara viticola: Dual-RNA-Seq Analysis Highlights Gene Expression Dynamics in The Pathogen and The Plant During The Battle For Infection
Source: Genes (Basel). 2020 Feb 28;11(3):261. doi: 10.3390/genes11030261 (PMC7140796; doi:10.3390/genes11030261)
Supplement: Supplementary file 1 [file genes-11-00261-s001.zip › Table S4.docx]

# **Table S4.** Enriched pathway terms in modules detected *via* the network analysis represented in Figure 4.

| **Module** | **Pathway** | **ID** | **Input** | **Background** | ***p*-Value** | **Corrected *p*-Value** |
| --- | --- | --- | --- | --- | --- | --- |
| **Green** | Sesquiterpenoid and triterpenoid biosynthesis | vvi00909 | 2 | 24 | 0.00622 | 0.061651513 |
|  | Monoterpenoid biosynthesis | vvi00902 | 2 | 27 | 0.00771 | 0.061651513 |
|  | Glutathione metabolism | vvi00480 | 3 | 124 | 0.02042 | 0.108883384 |
| **Black** | Sesquiterpenoid and triterpenoid biosynthesis | vvi00909 | 3 | 24 | 9.36E-05 | 0.001591591 |
|  | alpha-Linolenic acid metabolism | vvi00592 | 3 | 62 | 0.00128 | 0.010863879 |
|  | Biosynthesis of secondary metabolites | vvi01110 | 9 | 1182 | 0.01592 | 0.090212776 |
|  | Flavonoid biosynthesis | vvi00941 | 2 | 84 | 0.03241 | 0.111892613 |
|  | Protein processing in endoplasmic reticulum | vvi04141 | 3 | 210 | 0.03291 | 0.111892613 |
| **Blue** | Protein processing in endoplasmic reticulum | vvi04141 | 18 | 210 | 3.53E-10 | 1.69E-08 |
|  | Glucosinolate biosynthesis | vvi00966 | 5 | 10 | 6.45E-07 | 1.55E-05 |
|  | Cyanoamino acid metabolism | vvi00460 | 8 | 65 | 2.62E-06 | 4.20E-05 |
|  | Galactose metabolism | vvi00052 | 5 | 57 | 0.00088 | 0.010534596 |
|  | 2-Oxocarboxylic acid metabolism | vvi01210 | 5 | 61 | 0.00117 | 0.011196054 |
|  | Thiamine metabolism | vvi00730 | 3 | 17 | 0.00166 | 0.013280685 |
|  | Biosynthesis of secondary metabolites | vvi01110 | 26 | 1182 | 0.00296 | 0.020273217 |
|  | Plant-pathogen interaction | vvi04626 | 7 | 242 | 0.03081 | 0.18483309 |
|  | Amino sugar and nucleotide sugar metabolism | vvi00520 | 5 | 147 | 0.03626 | 0.193395439 |
|  | Glycine, serine and threonine metabolism | vvi00260 | 3 | 68 | 0.0534 | 0.241096133 |
|  | Circadian rhythm - plant | vvi04712 | 3 | 69 | 0.05525 | 0.241096133 |
| **Brown** | Flavone and flavonol biosynthesis | vvi00944 | 6 | 23 | 2.59E-06 | 0.00011121 |
|  | Flavonoid biosynthesis | vvi00941 | 7 | 84 | 0.00029 | 0.006248858 |
|  | AGE-RAGE signaling pathway in diabetic complications | vvi04933 | 3 | 17 | 0.00258 | 0.023732911 |
|  | Phagosome | vvi04145 | 6 | 93 | 0.00269 | 0.023732911 |
|  | Steroid biosynthesis | vvi00100 | 4 | 38 | 0.00276 | 0.023732911 |
|  | Protein processing in endoplasmic reticulum | vvi04141 | 9 | 210 | 0.00384 | 0.027509032 |
|  | DNA replication | vvi03030 | 4 | 49 | 0.0064 | 0.036754779 |
|  | Cutin, suberine and wax biosynthesis | vvi00073 | 3 | 25 | 0.00684 | 0.036754779 |
| **Pink** | Linoleic acid metabolism | vvi00591 | 2 | 17 | 0.00207 | 0.028958481 |
|  | Amino sugar and nucleotide sugar metabolism | vvi00520 | 3 | 147 | 0.01661 | 0.102581441 |
|  | alpha-Linolenic acid metabolism | vvi00592 | 2 | 62 | 0.02198 | 0.102581441 |
| **Magneta** | Glutathione metabolism | vvi00480 | 3 | 124 | 0.00033 | 0.001338747 |
| **Red** | Plant hormone signal transduction | vvi04075 | 7 | 280 | 0.00067 | 0.016755317 |
| **Turquoise** | Phenylpropanoid biosynthesis | vvi00940 | 20 | 196 | 1.25E-05 | 0.000775912 |
|  | Flavonoid biosynthesis | vvi00941 | 11 | 84 | 0.00016 | 0.004933944 |
|  | Biosynthesis of secondary metabolites | vvi01110 | 59 | 1182 | 0.00062 | 0.012502052 |
|  | Pentose and glucuronate interconversions | vvi00040 | 10 | 89 | 0.00093 | 0.012502052 |
|  | Glycerophospholipid metabolism | vvi00564 | 10 | 90 | 0.00101 | 0.012502052 |
|  | Starch and sucrose metabolism | vvi00500 | 16 | 225 | 0.00332 | 0.034347141 |
|  | Metabolic pathways | vvi01100 | 80 | 2006 | 0.02301 | 0.180775629 |
|  | Amino sugar and nucleotide sugar metabolism | vvi00520 | 10 | 147 | 0.02333 | 0.180775629 |
|  | Photosynthesis - antenna proteins | vvi00196 | 3 | 19 | 0.02871 | 0.197777193 |
|  | Sesquiterpenoid and triterpenoid biosynthesis | vvi00909 | 3 | 24 | 0.04875 | 0.300971293 |
|  | Cutin, suberine and wax biosynthesis | vvi00073 | 3 | 25 | 0.0534 | 0.300971293 |
| **Yellow** | Tropane, piperidine and pyridine alkaloid biosynthesis | vvi00960 | 3 | 33 | 0.00136 | 0.028650913 |
|  | Plant-pathogen interaction | vvi04626 | 6 | 242 | 0.00422 | 0.044295021 |
|  | Glutathione metabolism | vvi00480 | 4 | 124 | 0.00787 | 0.055117283 |
|  | Monoterpenoid biosynthesis | vvi00902 | 2 | 27 | 0.01342 | 0.070476832 |
