# Supplementary material for: Novel Aspects on The Interaction Between Grapevine and Plasmopara viticola: Dual-RNA-Seq Analysis Highlights Gene Expression Dynamics in The Pathogen and The Plant During The Battle For Infection
Source: Genes (Basel). 2020 Feb 28;11(3):261. doi: 10.3390/genes11030261 (PMC7140796; doi:10.3390/genes11030261)
Supplement: Supplementary file 1 [file genes-11-00261-s001.zip › Table S5.docx]

**Table S5.** List of transcription factors (TFs) detected in each module of network analysis represented in Figure 4.

| **Module** | **Gene ID** | **TF Name** |
| --- | --- | --- |
| **Green** | XP_002264229.1, XP_002262889.2 | ERF, MIKC_MADS |
| **Black** | XP_002276877.1, XP_010653965.1 | FAR1, bHLH |
| **Blue** | XP_002275182.2, XP_010658439.1, XP_010661963.2, XP_002264523.1, XP_002264656.1, XP_002267961.1, XP_002276153.1, XP_002276572.2, XP_002280097.1, XP_002282169.1, XP_002280048.1, XP_002275926.1, XP_003631256.1, XP_002282280.1 | LBD, LBD, LBD, ERF, ERF, ERF, ERF, ERF, ERF, ERF, HD-ZIP, C3H, MYB, MYB_related |
| **Brown:** | XP_002266233.1, XP_002267745.1, XP_002271458.1, XP_002276880.1, XP_002277678.1, XP_002278709.1, XP_002281083.2, XP_002281371.1, XP_002282566.1, XP_010648342.1, XP_010649949.1, XP_010649950.1, XP_010647760.1, XP_002272426.1, XP_010654438.1, XP_010654439.1, XP_010654440.1, XP_010662088.1, XP_002281033.1, XP_002274948.1, NP_001267930.1, XP_002271862.1, XP_002274992.2, XP_010660318.1 | YABBY, bHLH, NAC, GRAS, bHLH, HSF, bHLH, ZF-HD, NAC, HSF, CPP, CPP, B3, ERF, GRF, GRF, GRF, GRF, TALE, C3H, MYB, MYB, MYB, MYB |
| **Pink** | XP_002278333.1, XP_010647474.1, XP_010651405.1, XP_010651406.1, XP_010649137.1 | GRAS, bHLH, G2-like, G2-like, MYB_related |
| **Red** | XP_002284654.2, XP_002284668.1, XP_003632455.1, XP_010654080.1, XP_002282253.1, XP_010647274.1, XP_002268475.2, XP_002273805.4 | NAC, NAC, Dof, bZIP, ERF, ERF, G2-like, TALE |
| **Turquoise** | XP_002262764.1, XP_002267333.1, XP_002267819.2, XP_002273502.1, XP_002275319.1, XP_002275912.1, XP_002278824.1, XP_002279517.3, XP_002279929.1, XP_002282602.1, XP_002283811.1, XP_002284028.1, XP_002284836.1, XP_002285311.2, XP_003633906.1, XP_010648650.1, XP_010649545.1, XP_010653170.1, XP_010653274.1, XP_010661346.1, XP_010661504.1, XP_010663930.1, XP_010663931.1, XP_002272159.1, XP_002268413.2, XP_002280555.2, XP_002283864.1, XP_002285107.1, XP_010656426.1, XP_010660119.1, XP_010660384.1, XP_002270427.2, XP_002281639.1, XP_010663679.1, XP_010648331.1, XP_010661046.1, XP_002281099.1, XP_002284394.3, XP_010644321.1, XP_010663767.1, XP_010663776.1, NP_001267953.1, NP_001268160.1, NP_001268180.2, XP_002266427.1, XP_002279128.2, XP_002279874.1, XP_002281027.1, XP_010648383.1, XP_010661716.1 | bHLH, NAC, bHLH, GATA, NAC, bZIP, bHLH, GRAS, Trihelix, LBD, NAC, GATA, HSF, bZIP, SBP, bZIP, GATA, ZF-HD, bHLH, SBP, bHLH, NAC, NAC, AP2, ERF, ERF, ERF, ERF, ERF, ERF, ERF, GRF, GRF, WOX, TALE, HD-ZIP, LSD, C2H2, C2H2, LSD, LSD, MYB, MYB, MYB, MYB, MYB, MYB, MYB, MYB, MYB |
| **Yellow** | XP_010655428.2, XP_010664911.1, XP_003631660.1, XP_010662108.1 | HSF, MYB, MYB_related, MYB_related |
