# Supplementary material for: Novel Aspects on The Interaction Between Grapevine and Plasmopara viticola: Dual-RNA-Seq Analysis Highlights Gene Expression Dynamics in The Pathogen and The Plant During The Battle For Infection
Source: Genes (Basel). 2020 Feb 28;11(3):261. doi: 10.3390/genes11030261 (PMC7140796; doi:10.3390/genes11030261)
Supplement: Supplementary file 1 [file genes-11-00261-s001.zip › Table S6.docx]

**Table S6.** Transcription factors (TFs) enrichment of module genes detected via network analysis represented in Figure 4.

| **Module** | **Name** | **TF** | **Background_bind** | **Query_all** | **Query_bind** | ***p*_value** | ***q*_value** |
| --- | --- | --- | --- | --- | --- | --- | --- |
| **Black** | HSF | GSVIVG01019829001 | 506 | 33 | 3 | 3.539e-03 | 1.108e-01 |
|  | ERF | GSVIVG01022076001 | 842 | 33 | 4 | 3.720e-03 | 1.108e-01 |
|  | WRKY | GSVIVG01018300001 | 265 | 33 | 2 | 4.393e-03 | 1.108e-01 |
|  | HSF | GSVIVG01003118001 | 558 | 33 | 3 | 5.006e-03 | 1.108e-01 |
|  | HSF | GSVIVG01009477001 | 563 | 33 | 3 | 5.165e-03 | 1.108e-01 |
|  | bHLH | GSVIVG01018165001 | 571 | 33 | 3 | 5.428e-03 | 1.108e-01 |
|  | HD-ZIP | GSVIVG01020033001 | 294 | 33 | 2 | 5.859e-03 | 1.108e-01 |
|  | GATA | GSVIVG01035048001 | 962 | 33 | 4 | 6.518e-03 | 1.108e-01 |
|  | NAC | GSVIVG01014287001 | 338 | 33 | 2 | 8.589e-03 | 1.235e-01 |
|  | NAC | GSVIVG01013419001 | 345 | 33 | 2 | 9.081e-03 | 1.235e-01 |
|  | bHLH | GSVIVG01009292001 | 1204 | 33 | 4 | 1.617e-02 | 1.550e-01 |
|  | NAC | GSVIVG01029392001 | 449 | 33 | 2 | 1.837e-02 | 1.550e-01 |
|  | NAC | GSVIVG01013671001 | 479 | 33 | 2 | 2.176e-02 | 1.550e-01 |
|  | NAC | GSVIVG01026495001 | 538 | 33 | 2 | 2.936e-02 | 1.550e-01 |
|  | GATA | GSVIVG01029593001 | 1924 | 33 | 5 | 3.032e-02 | 1.550e-01 |
|  | NAC | GSVIVG01035214001 | 570 | 33 | 2 | 3.400e-02 | 1.550e-01 |
|  | NAC | GSVIVG01016176001 | 574 | 33 | 2 | 3.461e-02 | 1.550e-01 |
|  | MYB | GSVIVG01011417001 | 2007 | 33 | 5 | 3.628e-02 | 1.550e-01 |
|  | NAC | GSVIVG01014405001 | 590 | 33 | 2 | 3.709e-02 | 1.550e-01 |
|  | C2H2 | GSVIVG01010283001 | 610 | 33 | 2 | 4.031e-02 | 1.550e-01 |
|  | ARF | GSVIVG01025691001 | 613 | 33 | 2 | 4.081e-02 | 1.550e-01 |
|  | bZIP | GSVIVG01021790001 | 1050 | 33 | 3 | 4.103e-02 | 1.550e-01 |
|  | MYB | GSVIVG01029904001 | 1596 | 33 | 4 | 4.683e-02 | 1.592e-01 |
|  | C2H2 | GSVIVG01034505001 | 659 | 33 | 2 | 4.880e-02 | 1.619e-01 |
| **Blue** | HSF | GSVIVG01019829001 | 506 | 117 | 11 | 2.732e-06 | 6.584e-04 |
|  | bHLH | GSVIVG01028516001 | 1369 | 117 | 17 | 3.389e-05 | 3.211e-03 |
|  | HSF | GSVIVG01003118001 | 558 | 117 | 10 | 3.997e-05 | 3.211e-03 |
|  | MYB_related | GSVIVG01015223001 | 328 | 117 | 7 | 1.115e-04 | 5.829e-03 |
|  | bZIP | GSVIVG01009485001 | 1512 | 117 | 17 | 1.209e-04 | 5.829e-03 |
|  | TCP | GSVIVG01027588001 | 1574 | 117 | 17 | 1.991e-04 | 7.828e-03 |
|  | TCP | GSVIVG01014236001 | 1046 | 117 | 13 | 2.274e-04 | 7.828e-03 |
|  | TCP | GSVIVG01008023001 | 1469 | 117 | 16 | 2.654e-04 | 7.994e-03 |
|  | MYB | GSVIVG01008241001 | 489 | 117 | 8 | 3.414e-04 | 9.143e-03 |
|  | MYB_related | GSVIVG01013871001 | 397 | 117 | 7 | 4.049e-04 | 9.759e-03 |
|  | WRKY | GSVIVG01021252001 | 229 | 117 | 5 | 5.661e-04 | 1.240e-02 |
|  | C2H2 | GSVIVG01025855001 | 6728 | 117 | 45 | 7.197e-04 | 1.391e-02 |
|  | ERF | GSVIVG01009007001 | 784 | 117 | 10 | 7.501e-04 | 1.391e-02 |
|  | NAC | GSVIVG01029392001 | 449 | 117 | 7 | 9.038e-04 | 1.556e-02 |
|  | HSF | GSVIVG01015389001 | 464 | 117 | 7 | 1.115e-03 | 1.766e-02 |
|  | bZIP | GSVIVG01001940001 | 1526 | 117 | 15 | 1.173e-03 | 1.766e-02 |
|  | WRKY | GSVIVG01021765001 | 273 | 117 | 5 | 1.403e-03 | 1.976e-02 |
|  | MYB_related | GSVIVG01025544001 | 485 | 117 | 7 | 1.476e-03 | 1.976e-02 |
|  | MYB_related | GSVIVG01005816001 | 391 | 117 | 6 | 1.843e-03 | 2.338e-02 |
|  | bZIP | GSVIVG01013053001 | 1612 | 117 | 15 | 2.057e-03 | 2.479e-02 |
|  | MYB_related | GSVIVG01020132001 | 308 | 117 | 5 | 2.571e-03 | 2.950e-02 |
|  | NAC | GSVIVG01015274001 | 545 | 117 | 7 | 3.034e-03 | 3.125e-02 |
|  | MYB_related | GSVIVG01035231001 | 429 | 117 | 6 | 3.091e-03 | 3.125e-02 |
|  | TCP | GSVIVG01026145001 | 1527 | 117 | 14 | 3.145e-03 | 3.125e-02 |
|  | Trihelix | GSVIVG01011168001 | 678 | 117 | 8 | 3.297e-03 | 3.125e-02 |
|  | MYB | GSVIVG01020038001 | 949 | 117 | 10 | 3.372e-03 | 3.125e-02 |
|  | HSF | GSVIVG01009477001 | 563 | 117 | 7 | 3.691e-03 | 3.295e-02 |
|  | bZIP | GSVIVG01023817001 | 1005 | 117 | 10 | 5.168e-03 | 4.364e-02 |
|  | WRKY | GSVIVG01028244001 | 252 | 117 | 4 | 5.410e-03 | 4.364e-02 |
|  | C2H2 | GSVIVG01010283001 | 610 | 117 | 7 | 5.933e-03 | 4.364e-02 |
|  | bZIP | GSVIVG01019009001 | 1481 | 117 | 13 | 6.033e-03 | 4.364e-02 |
|  | C2H2 | GSVIVG01016867001 | 746 | 117 | 8 | 6.105e-03 | 4.364e-02 |
|  | C2H2 | GSVIVG01035391001 | 746 | 117 | 8 | 6.105e-03 | 4.364e-02 |
|  | C2H2 | GSVIVG01019000001 | 747 | 117 | 8 | 6.157e-03 | 4.364e-02 |
|  | G2-like | GSVIVG01003551001 | 262 | 117 | 4 | 6.358e-03 | 4.378e-02 |
|  | TCP | GSVIVG01036449001 | 1830 | 117 | 15 | 7.020e-03 | 4.505e-02 |
|  | MYB | GSVIVG01034001001 | 500 | 117 | 6 | 7.042e-03 | 4.505e-02 |
|  | bZIP | GSVIVG01021790001 | 1050 | 117 | 10 | 7.103e-03 | 4.505e-02 |
|  | C2H2 | GSVIVG01010284001 | 911 | 117 | 9 | 7.443e-03 | 4.599e-02 |
|  | bZIP | GSVIVG01031238001 | 921 | 117 | 9 | 8.006e-03 | 4.824e-02 |
|  | bZIP | GSVIVG01033832001 | 1536 | 117 | 13 | 8.211e-03 | 4.827e-02 |
|  | WRKY | GSVIVG01026969001 | 287 | 117 | 4 | 9.225e-03 | 5.152e-02 |
|  | NAC | GSVIVG01016175001 | 527 | 117 | 6 | 9.256e-03 | 5.152e-02 |
|  | HD-ZIP | GSVIVG01038619001 | 530 | 117 | 6 | 9.530e-03 | 5.152e-02 |
|  | NAC | GSVIVG01033372001 | 290 | 117 | 4 | 9.620e-03 | 5.152e-02 |
|  | HD-ZIP | GSVIVG01020033001 | 294 | 117 | 4 | 1.017e-02 | 5.326e-02 |
|  | MYB | GSVIVG01019967001 | 105 | 117 | 2 | 1.152e-02 | 5.908e-02 |
|  | NAC | GSVIVG01000940001 | 198 | 117 | 3 | 1.187e-02 | 5.943e-02 |
|  | MYB | GSVIVG01036090001 | 307 | 117 | 4 | 1.208e-02 | 5.943e-02 |
|  | bHLH | GSVIVG01008164001 | 839 | 117 | 8 | 1.258e-02 | 6.064e-02 |
|  | WRKY | GSVIVG01012196001 | 205 | 117 | 3 | 1.334e-02 | 6.204e-02 |
|  | bZIP | GSVIVG01027040001 | 1467 | 117 | 12 | 1.339e-02 | 6.204e-02 |
|  | WRKY | GSVIVG01026965001 | 320 | 117 | 4 | 1.423e-02 | 6.472e-02 |
|  | ZF-HD | GSVIVG01018947001 | 324 | 117 | 4 | 1.494e-02 | 6.669e-02 |
|  | WRKY | GSVIVG01029265001 | 334 | 117 | 4 | 1.682e-02 | 7.370e-02 |
|  | BES1 | GSVIVG01036911001 | 737 | 117 | 7 | 1.714e-02 | 7.376e-02 |
|  | NAC | GSVIVG01014287001 | 338 | 117 | 4 | 1.761e-02 | 7.447e-02 |
|  | WRKY | GSVIVG01010525001 | 340 | 117 | 4 | 1.802e-02 | 7.487e-02 |
|  | NAC | GSVIVG01019952001 | 229 | 117 | 3 | 1.922e-02 | 7.853e-02 |
|  | WRKY | GSVIVG01032662001 | 235 | 117 | 3 | 2.091e-02 | 8.399e-02 |
|  | NAC | GSVIVG01020609001 | 623 | 117 | 6 | 2.131e-02 | 8.421e-02 |
|  | MIKC_MADS | GSVIVG01001437001 | 631 | 117 | 6 | 2.266e-02 | 8.808e-02 |
|  | C2H2 | GSVIVG01020612001 | 787 | 117 | 7 | 2.425e-02 | 9.132e-02 |
|  | WRKY | GSVIVG01024624001 | 369 | 117 | 4 | 2.461e-02 | 9.132e-02 |
|  | bZIP | GSVIVG01010152001 | 942 | 117 | 8 | 2.463e-02 | 9.132e-02 |
|  | bZIP | GSVIVG01035829001 | 795 | 117 | 7 | 2.556e-02 | 9.332e-02 |
|  | WRKY | GSVIVG01009441001 | 375 | 117 | 4 | 2.614e-02 | 9.404e-02 |
|  | bZIP | GSVIVG01013443001 | 147 | 117 | 2 | 2.790e-02 | 9.890e-02 |
|  | WRKY | GSVIVG01000752001 | 388 | 117 | 4 | 2.967e-02 | 1.029e-01 |
|  | ERF | GSVIVG01015037001 | 1300 | 117 | 10 | 2.994e-02 | 1.029e-01 |
|  | ARF | GSVIVG01009865001 | 264 | 117 | 3 | 3.033e-02 | 1.029e-01 |
|  | WRKY | GSVIVG01012682001 | 268 | 117 | 3 | 3.179e-02 | 1.064e-01 |
|  | NAC | GSVIVG01034485001 | 271 | 117 | 3 | 3.292e-02 | 1.087e-01 |
|  | ERF | GSVIVG01022076001 | 842 | 117 | 7 | 3.424e-02 | 1.106e-01 |
|  | ERF | GSVIVG01002195001 | 692 | 117 | 6 | 3.489e-02 | 1.106e-01 |
|  | ERF | GSVIVG01006277001 | 692 | 117 | 6 | 3.489e-02 | 1.106e-01 |
|  | G2-like | GSVIVG01024916001 | 162 | 117 | 2 | 3.569e-02 | 1.117e-01 |
|  | bHLH | GSVIVG01010100001 | 1014 | 117 | 8 | 3.690e-02 | 1.140e-01 |
|  | HD-ZIP | GSVIVG01027407001 | 417 | 117 | 4 | 3.860e-02 | 1.177e-01 |
|  | WRKY | GSVIVG01027069001 | 289 | 117 | 3 | 4.016e-02 | 1.210e-01 |
|  | bHLH | GSVIVG01027766001 | 717 | 117 | 6 | 4.098e-02 | 1.216e-01 |
|  | WRKY | GSVIVG01021397001 | 426 | 117 | 4 | 4.167e-02 | 1.216e-01 |
|  | NAC | GSVIVG01035214001 | 570 | 117 | 5 | 4.192e-02 | 1.216e-01 |
|  | ERF | GSVIVG01031387001 | 428 | 117 | 4 | 4.237e-02 | 1.216e-01 |
|  | NAC | GSVIVG01001264001 | 299 | 117 | 3 | 4.455e-02 | 1.249e-01 |
|  | ARF | GSVIVG01008639001 | 299 | 117 | 3 | 4.455e-02 | 1.249e-01 |
|  | G2-like | GSVIVG01022645001 | 308 | 117 | 3 | 4.872e-02 | 1.350e-01 |
|  | bHLH | GSVIVG01031338001 | 1240 | 117 | 9 | 4.939e-02 | 1.353e-01 |
| **Brown** | MYB | GSVIVG01035664001 | 330 | 156 | 26 | 4.841e-23 | 1.123e-20 |
|  | BBR-BPC | GSVIVG01009845001 | 5654 | 156 | 68 | 1.541e-10 | 1.788e-08 |
|  | BBR-BPC | GSVIVG01011589001 | 7411 | 156 | 75 | 3.831e-08 | 2.962e-06 |
|  | GRAS | GSVIVG01011710001 | 6537 | 156 | 67 | 2.201e-07 | 1.276e-05 |
|  | AP2 | GSVIVG01019049001 | 9679 | 156 | 82 | 1.933e-05 | 8.968e-04 |
|  | C2H2 | GSVIVG01025855001 | 6728 | 156 | 62 | 3.321e-05 | 1.268e-03 |
|  | MIKC_MADS | GSVIVG01027579001 | 10323 | 156 | 85 | 3.826e-05 | 1.268e-03 |
|  | TCP | GSVIVG01036449001 | 1830 | 156 | 23 | 1.974e-04 | 5.726e-03 |
|  | MYB | GSVIVG01034041001 | 167 | 156 | 5 | 4.919e-04 | 1.268e-02 |
|  | NAC | GSVIVG01016176001 | 574 | 156 | 10 | 6.469e-04 | 1.501e-02 |
|  | TALE | GSVIVG01009273001 | 5809 | 156 | 51 | 7.590e-04 | 1.591e-02 |
|  | C2H2 | GSVIVG01016182001 | 1105 | 156 | 15 | 8.914e-04 | 1.591e-02 |
|  | C2H2 | GSVIVG01031461001 | 1105 | 156 | 15 | 8.914e-04 | 1.591e-02 |
|  | GATA | GSVIVG01009197001 | 938 | 156 | 13 | 1.415e-03 | 2.344e-02 |
|  | NAC | GSVIVG01033374001 | 293 | 156 | 6 | 1.864e-03 | 2.705e-02 |
|  | HSF | GSVIVG01003118001 | 558 | 156 | 9 | 1.865e-03 | 2.705e-02 |
|  | MYB | GSVIVG01008484001 | 91 | 156 | 3 | 2.120e-03 | 2.893e-02 |
|  | HSF | GSVIVG01019829001 | 506 | 156 | 8 | 3.285e-03 | 4.234e-02 |
|  | MYB | GSVIVG01019967001 | 105 | 156 | 3 | 3.557e-03 | 4.344e-02 |
|  | NAC | GSVIVG01036711001 | 435 | 156 | 7 | 4.505e-03 | 4.994e-02 |
|  | bHLH | GSVIVG01019659001 | 344 | 156 | 6 | 4.521e-03 | 4.994e-02 |
|  | NAC | GSVIVG01015274001 | 545 | 156 | 8 | 5.309e-03 | 5.598e-02 |
|  | bZIP | GSVIVG01027040001 | 1467 | 156 | 16 | 6.263e-03 | 5.667e-02 |
|  | TCP | GSVIVG01008023001 | 1469 | 156 | 16 | 6.345e-03 | 5.667e-02 |
|  | NAC | GSVIVG01008839001 | 278 | 156 | 5 | 6.368e-03 | 5.667e-02 |
|  | HSF | GSVIVG01009477001 | 563 | 156 | 8 | 6.520e-03 | 5.667e-02 |
|  | NAC | GSVIVG01000940001 | 198 | 156 | 4 | 6.595e-03 | 5.667e-02 |
|  | E2F/DP | GSVIVG01009890001 | 474 | 156 | 7 | 7.434e-03 | 6.160e-02 |
|  | NAC | GSVIVG01036682001 | 393 | 156 | 6 | 9.123e-03 | 7.298e-02 |
|  | bHLH | GSVIVG01027766001 | 717 | 156 | 9 | 1.055e-02 | 8.158e-02 |
|  | MYB | GSVIVG01020038001 | 949 | 156 | 11 | 1.103e-02 | 8.255e-02 |
|  | NAC | GSVIVG01023123001 | 319 | 156 | 5 | 1.204e-02 | 8.281e-02 |
|  | TCP | GSVIVG01027588001 | 1574 | 156 | 16 | 1.212e-02 | 8.281e-02 |
|  | NAC | GSVIVG01020609001 | 623 | 156 | 8 | 1.214e-02 | 8.281e-02 |
|  | NAC | GSVIVG01026495001 | 538 | 156 | 7 | 1.508e-02 | 9.996e-02 |
|  | TCP | GSVIVG01032911001 | 1000 | 156 | 11 | 1.607e-02 | 1.035e-01 |
|  | NAC | GSVIVG01013419001 | 345 | 156 | 5 | 1.709e-02 | 1.071e-01 |
|  | NAC | GSVIVG01029392001 | 449 | 156 | 6 | 1.781e-02 | 1.087e-01 |
|  | TCP | GSVIVG01026145001 | 1527 | 156 | 15 | 1.912e-02 | 1.138e-01 |
|  | C2H2 | GSVIVG01010284001 | 911 | 156 | 10 | 1.996e-02 | 1.146e-01 |
|  | bZIP | GSVIVG01035829001 | 795 | 156 | 9 | 2.025e-02 | 1.146e-01 |
|  | TCP | GSVIVG01014236001 | 1046 | 156 | 11 | 2.196e-02 | 1.213e-01 |
|  | LBD | GSVIVG01027621001 | 818 | 156 | 9 | 2.407e-02 | 1.299e-01 |
|  | bZIP | GSVIVG01010152001 | 942 | 156 | 10 | 2.476e-02 | 1.306e-01 |
|  | Trihelix | GSVIVG01016703001 | 710 | 156 | 8 | 2.584e-02 | 1.332e-01 |
|  | bHLH | GSVIVG01023585001 | 603 | 156 | 7 | 2.752e-02 | 1.340e-01 |
|  | AP2 | GSVIVG01004382001 | 288 | 156 | 4 | 2.872e-02 | 1.340e-01 |
|  | WRKY | GSVIVG01027069001 | 289 | 156 | 4 | 2.909e-02 | 1.340e-01 |
|  | C2H2 | GSVIVG01010283001 | 610 | 156 | 7 | 2.919e-02 | 1.340e-01 |
|  | TCP | GSVIVG01008109001 | 967 | 156 | 10 | 2.922e-02 | 1.340e-01 |
|  | NAC | GSVIVG01033372001 | 290 | 156 | 4 | 2.946e-02 | 1.340e-01 |
|  | NAC | GSVIVG01008291001 | 299 | 156 | 4 | 3.297e-02 | 1.443e-01 |
|  | ARF | GSVIVG01008639001 | 299 | 156 | 4 | 3.297e-02 | 1.443e-01 |
|  | GATA | GSVIVG01017011001 | 210 | 156 | 3 | 3.653e-02 | 1.563e-01 |
|  | bZIP | GSVIVG01023817001 | 1005 | 156 | 10 | 3.705e-02 | 1.563e-01 |
|  | NAC | GSVIVG01016175001 | 527 | 156 | 6 | 3.784e-02 | 1.568e-01 |
|  | HD-ZIP | GSVIVG01017010001 | 320 | 156 | 4 | 4.212e-02 | 1.698e-01 |
|  | ERF | GSVIVG01031387001 | 428 | 156 | 5 | 4.246e-02 | 1.698e-01 |
|  | MYB | GSVIVG01025269001 | 2390 | 156 | 20 | 4.366e-02 | 1.702e-01 |
|  | ZF-HD | GSVIVG01018947001 | 324 | 156 | 4 | 4.402e-02 | 1.702e-01 |
|  | Dof | GSVIVG01016887001 | 5391 | 156 | 40 | 4.691e-02 | 1.784e-01 |
| **Green** | MYB | GSVIVG01008241001 | 489 | 23 | 3 | 7.844e-04 | 6.230e-02 |
|  | G2-like | GSVIVG01007065001 | 228 | 23 | 2 | 9.968e-04 | 6.230e-02 |
|  | G2-like | GSVIVG01022645001 | 308 | 23 | 2 | 2.356e-03 | 7.982e-02 |
|  | MYB_related | GSVIVG01015223001 | 328 | 23 | 2 | 2.815e-03 | 7.982e-02 |
|  | G2-like | GSVIVG01011942001 | 361 | 23 | 2 | 3.686e-03 | 7.982e-02 |
|  | G2-like | GSVIVG01033381001 | 366 | 23 | 2 | 3.831e-03 | 7.982e-02 |
|  | MYB_related | GSVIVG01005816001 | 391 | 23 | 2 | 4.608e-03 | 8.228e-02 |
|  | MYB_related | GSVIVG01025544001 | 485 | 23 | 2 | 8.350e-03 | 1.160e-01 |
|  | ERF | GSVIVG01025461001 | 1725 | 23 | 4 | 1.493e-02 | 1.518e-01 |
|  | ERF | GSVIVG01002195001 | 692 | 23 | 2 | 2.161e-02 | 1.589e-01 |
|  | ERF | GSVIVG01006277001 | 692 | 23 | 2 | 2.161e-02 | 1.589e-01 |
|  | ERF | GSVIVG01015037001 | 1300 | 23 | 3 | 2.467e-02 | 1.666e-01 |
|  | C2H2 | GSVIVG01025855001 | 6728 | 23 | 9 | 4.656e-02 | 2.155e-01 |
| **Pink** | NAC | GSVIVG01035214001 | 570 | 13 | 3 | 1.327e-04 | 6.901e-03 |
|  | MYB | GSVIVG01029904001 | 1596 | 13 | 4 | 6.921e-04 | 1.356e-02 |
|  | bZIP | GSVIVG01010836001 | 383 | 13 | 2 | 7.823e-04 | 1.356e-02 |
|  | NAC | GSVIVG01036711001 | 435 | 13 | 2 | 1.130e-03 | 1.469e-02 |
|  | MYB | GSVIVG01011417001 | 2007 | 13 | 4 | 1.954e-03 | 1.757e-02 |
|  | NAC | GSVIVG01026495001 | 538 | 13 | 2 | 2.079e-03 | 1.757e-02 |
|  | NAC | GSVIVG01016176001 | 574 | 13 | 2 | 2.500e-03 | 1.757e-02 |
|  | NAC | GSVIVG01014405001 | 590 | 13 | 2 | 2.703e-03 | 1.757e-02 |
|  | NAC | GSVIVG01013182001 | 770 | 13 | 2 | 5.712e-03 | 2.970e-02 |
|  | Dof | GSVIVG01008156001 | 1633 | 13 | 3 | 6.689e-03 | 3.162e-02 |
|  | MYB | GSVIVG01036712001 | 1192 | 13 | 2 | 1.880e-02 | 6.075e-02 |
|  | C2H2 | GSVIVG01007564001 | 1217 | 13 | 2 | 1.986e-02 | 6.075e-02 |
|  | Nin-like | GSVIVG01013370001 | 1426 | 13 | 2 | 3.009e-02 | 7.381e-02 |
|  | ERF | GSVIVG01025461001 | 1725 | 13 | 2 | 4.888e-02 | 9.219e-02 |
| **Red** | C2H2 | GSVIVG01025855001 | 6728 | 53 | 24 | 5.435e-04 | 9.892e-02 |
|  | bHLH | GSVIVG01019659001 | 344 | 53 | 3 | 5.056e-03 | 2.459e-01 |
|  | B3 | GSVIVG01033007001 | 603 | 53 | 4 | 7.176e-03 | 2.459e-01 |
|  | TCP | GSVIVG01026145001 | 1527 | 53 | 7 | 1.081e-02 | 2.459e-01 |
|  | G2-like | GSVIVG01007065001 | 228 | 53 | 2 | 1.090e-02 | 2.459e-01 |
|  | WRKY | GSVIVG01021252001 | 229 | 53 | 2 | 1.103e-02 | 2.459e-01 |
|  | WRKY | GSVIVG01037775001 | 231 | 53 | 2 | 1.129e-02 | 2.459e-01 |
|  | MYB | GSVIVG01004464001 | 1557 | 53 | 7 | 1.206e-02 | 2.459e-01 |
|  | TCP | GSVIVG01027588001 | 1574 | 53 | 7 | 1.282e-02 | 2.459e-01 |
|  | WRKY | GSVIVG01032661001 | 247 | 53 | 2 | 1.351e-02 | 2.459e-01 |
|  | bHLH | GSVIVG01010100001 | 1014 | 53 | 5 | 1.577e-02 | 2.461e-01 |
|  | WRKY | GSVIVG01021765001 | 273 | 53 | 2 | 1.761e-02 | 2.461e-01 |
|  | MIKC_MADS | GSVIVG01027577001 | 772 | 53 | 4 | 1.922e-02 | 2.461e-01 |
|  | GRAS | GSVIVG01011710001 | 6537 | 53 | 19 | 2.514e-02 | 2.461e-01 |
|  | HD-ZIP | GSVIVG01017010001 | 320 | 53 | 2 | 2.661e-02 | 2.461e-01 |
|  | WRKY | GSVIVG01026965001 | 320 | 53 | 2 | 2.661e-02 | 2.461e-01 |
|  | Nin-like | GSVIVG01019600001 | 565 | 53 | 3 | 2.681e-02 | 2.461e-01 |
|  | TCP | GSVIVG01008023001 | 1469 | 53 | 6 | 2.704e-02 | 2.461e-01 |
|  | bZIP | GSVIVG01001940001 | 1526 | 53 | 6 | 3.236e-02 | 2.537e-01 |
|  | bHLH | GSVIVG01009292001 | 1204 | 53 | 5 | 3.319e-02 | 2.537e-01 |
|  | GATA | GSVIVG01029593001 | 1924 | 53 | 7 | 3.737e-02 | 2.537e-01 |
|  | MYB | GSVIVG01023527001 | 372 | 53 | 2 | 3.894e-02 | 2.537e-01 |
|  | SRS | GSVIVG01018845001 | 380 | 53 | 2 | 4.105e-02 | 2.537e-01 |
|  | NAC | GSVIVG01018809001 | 386 | 53 | 2 | 4.268e-02 | 2.537e-01 |
|  | WRKY | GSVIVG01000752001 | 388 | 53 | 2 | 4.323e-02 | 2.537e-01 |
|  | ERF | GSVIVG01015037001 | 1300 | 53 | 5 | 4.551e-02 | 2.537e-01 |
|  | Trihelix | GSVIVG01011168001 | 678 | 53 | 3 | 4.725e-02 | 2.537e-01 |
|  | MYB | GSVIVG01035041001 | 407 | 53 | 2 | 4.862e-02 | 2.537e-01 |
| **Turquoise** | C2H2 | GSVIVG01025855001 | 6728 | 373 | 138 | 3.232e-07 | 7.366e-05 |
|  | GRAS | GSVIVG01011710001 | 6537 | 373 | 134 | 5.581e-07 | 7.366e-05 |
|  | BBR-BPC | GSVIVG01011589001 | 7411 | 373 | 146 | 1.471e-06 | 1.006e-04 |
|  | BBR-BPC | GSVIVG01009845001 | 5654 | 373 | 118 | 1.525e-06 | 1.006e-04 |
|  | ARF | GSVIVG01023149001 | 179 | 373 | 11 | 1.013e-05 | 5.350e-04 |
|  | MIKC_MADS | GSVIVG01027579001 | 10323 | 373 | 183 | 3.938e-05 | 1.733e-03 |
|  | MYB | GSVIVG01011417001 | 2007 | 373 | 49 | 6.919e-05 | 2.610e-03 |
|  | MYB | GSVIVG01029904001 | 1596 | 373 | 40 | 1.730e-04 | 5.711e-03 |
|  | MYB | GSVIVG01003966001 | 771 | 373 | 23 | 2.952e-04 | 8.659e-03 |
|  | MYB | GSVIVG01024036001 | 831 | 373 | 24 | 3.680e-04 | 9.716e-03 |
|  | MYB | GSVIVG01027811001 | 2515 | 373 | 55 | 4.572e-04 | 1.097e-02 |
|  | MYB | GSVIVG01031341001 | 1759 | 373 | 41 | 6.518e-04 | 1.354e-02 |
|  | MYB | GSVIVG01036802001 | 1381 | 373 | 34 | 6.666e-04 | 1.354e-02 |
|  | ERF | GSVIVG01002195001 | 692 | 373 | 20 | 9.599e-04 | 1.689e-02 |
|  | ERF | GSVIVG01006277001 | 692 | 373 | 20 | 9.599e-04 | 1.689e-02 |
|  | TALE | GSVIVG01009273001 | 5809 | 373 | 106 | 1.493e-03 | 2.463e-02 |
|  | MYB | GSVIVG01033670001 | 196 | 373 | 8 | 1.999e-03 | 3.105e-02 |
|  | MIKC_MADS | GSVIVG01012250001 | 790 | 373 | 21 | 2.164e-03 | 3.173e-02 |
|  | NAC | GSVIVG01035214001 | 570 | 373 | 16 | 3.394e-03 | 4.716e-02 |
|  | MYB | GSVIVG01004851001 | 1444 | 373 | 32 | 4.822e-03 | 6.103e-02 |
|  | MYB | GSVIVG01028091001 | 184 | 373 | 7 | 4.855e-03 | 6.103e-02 |
|  | MYB | GSVIVG01036712001 | 1192 | 373 | 27 | 6.405e-03 | 7.686e-02 |
|  | MYB | GSVIVG01004464001 | 1557 | 373 | 33 | 8.312e-03 | 9.003e-02 |
|  | ERF | GSVIVG01031387001 | 428 | 373 | 12 | 8.471e-03 | 9.003e-02 |
|  | NAC | GSVIVG01016175001 | 527 | 373 | 14 | 8.647e-03 | 9.003e-02 |
|  | bHLH | GSVIVG01008164001 | 839 | 373 | 20 | 8.867e-03 | 9.003e-02 |
|  | bHLH | GSVIVG01021032001 | 386 | 373 | 11 | 9.428e-03 | 9.218e-02 |
|  | AP2 | GSVIVG01019049001 | 9679 | 373 | 158 | 1.060e-02 | 9.994e-02 |
|  | ERF | GSVIVG01036389001 | 3293 | 373 | 61 | 1.156e-02 | 1.053e-01 |
|  | WRKY | GSVIVG01014854001 | 135 | 373 | 5 | 1.265e-02 | 1.113e-01 |
|  | MYB | GSVIVG01036552001 | 178 | 373 | 6 | 1.373e-02 | 1.143e-01 |
|  | MYB | GSVIVG01035041001 | 407 | 373 | 11 | 1.385e-02 | 1.143e-01 |
|  | GATA | GSVIVG01029593001 | 1924 | 373 | 38 | 1.531e-02 | 1.225e-01 |
|  | MYB | GSVIVG01026868001 | 1058 | 373 | 23 | 1.641e-02 | 1.274e-01 |
|  | MYB | GSVIVG01025269001 | 2390 | 373 | 45 | 2.038e-02 | 1.496e-01 |
|  | TCP | GSVIVG01021528001 | 482 | 373 | 12 | 2.073e-02 | 1.496e-01 |
|  | bHLH | GSVIVG01000771001 | 111 | 373 | 4 | 2.097e-02 | 1.496e-01 |
|  | Dof | GSVIVG01011138001 | 751 | 373 | 17 | 2.175e-02 | 1.511e-01 |
|  | NAC | GSVIVG01033372001 | 290 | 373 | 8 | 2.308e-02 | 1.562e-01 |
|  | SBP | GSVIVG01028208001 | 156 | 373 | 5 | 2.398e-02 | 1.583e-01 |
|  | NAC | GSVIVG01013419001 | 345 | 373 | 9 | 2.600e-02 | 1.674e-01 |
|  | HD-ZIP | GSVIVG01002447001 | 204 | 373 | 6 | 2.666e-02 | 1.676e-01 |
|  | NAC | GSVIVG01008291001 | 299 | 373 | 8 | 2.738e-02 | 1.681e-01 |
|  | ERF | GSVIVG01015037001 | 1300 | 373 | 26 | 3.085e-02 | 1.838e-01 |
|  | ERF | GSVIVG01028315001 | 1723 | 373 | 33 | 3.189e-02 | 1.838e-01 |
|  | ERF | GSVIVG01022076001 | 842 | 373 | 18 | 3.203e-02 | 1.838e-01 |
|  | MYB | GSVIVG01011872001 | 214 | 373 | 6 | 3.332e-02 | 1.872e-01 |
|  | WOX | GSVIVG01018787001 | 467 | 373 | 11 | 3.523e-02 | 1.902e-01 |
|  | NAC | GSVIVG01016176001 | 574 | 373 | 13 | 3.530e-02 | 1.902e-01 |
|  | Dof | GSVIVG01008156001 | 1633 | 373 | 31 | 3.975e-02 | 2.099e-01 |
|  | ERF | GSVIVG01032961001 | 2569 | 373 | 46 | 4.117e-02 | 2.102e-01 |
|  | NAC | GSVIVG01013671001 | 479 | 373 | 11 | 4.140e-02 | 2.102e-01 |
|  | TCP | GSVIVG01008023001 | 1469 | 373 | 28 | 4.511e-02 | 2.247e-01 |
|  | NAC | GSVIVG01015274001 | 545 | 373 | 12 | 4.800e-02 | 2.347e-01 |
|  | B3 | GSVIVG01033007001 | 603 | 373 | 13 | 4.957e-02 | 2.379e-01 |
| **Yellow** | WOX | GSVIVG01026638001 | 221 | 45 | 2 | 6.375e-03 | 2.771e-01 |
|  | NAC | GSVIVG01035554001 | 272 | 45 | 2 | 1.122e-02 | 2.771e-01 |
|  | ARF | GSVIVG01020805001 | 289 | 45 | 2 | 1.320e-02 | 2.771e-01 |
|  | NAC | GSVIVG01033372001 | 290 | 45 | 2 | 1.332e-02 | 2.771e-01 |
|  | NAC | GSVIVG01008291001 | 299 | 45 | 2 | 1.445e-02 | 2.771e-01 |
|  | NAC | GSVIVG01019670001 | 313 | 45 | 2 | 1.631e-02 | 2.771e-01 |
|  | NAC | GSVIVG01023123001 | 319 | 45 | 2 | 1.715e-02 | 2.771e-01 |
|  | MYB | GSVIVG01035664001 | 330 | 45 | 2 | 1.875e-02 | 2.771e-01 |
|  | G2-like | GSVIVG01011942001 | 361 | 45 | 2 | 2.369e-02 | 2.783e-01 |
|  | CPP | GSVIVG01015363001 | 393 | 45 | 2 | 2.946e-02 | 2.783e-01 |
|  | MYB | GSVIVG01035041001 | 407 | 45 | 2 | 3.220e-02 | 2.783e-01 |
|  | MYB_related | GSVIVG01035231001 | 429 | 45 | 2 | 3.676e-02 | 2.783e-01 |
|  | HSF | GSVIVG01015389001 | 464 | 45 | 2 | 4.468e-02 | 2.783e-01 |
|  | MIKC_MADS | GSVIVG01012250001 | 790 | 45 | 3 | 4.536e-02 | 2.783e-01 |
|  | C2H2 | GSVIVG01025855001 | 6728 | 45 | 16 | 4.730e-02 | 2.783e-01 |
|  | MYB_related | GSVIVG01025544001 | 485 | 45 | 2 | 4.980e-02 | 2.783e-01 |
