# Supplementary figures and images for: Novel Aspects on The Interaction Between Grapevine and Plasmopara viticola: Dual-RNA-Seq Analysis Highlights Gene Expression Dynamics in The Pathogen and The Plant During The Battle For Infection
Source: Genes (Basel). 2020 Feb 28;11(3):261. doi: 10.3390/genes11030261 (PMC7140796; doi:10.3390/genes11030261)

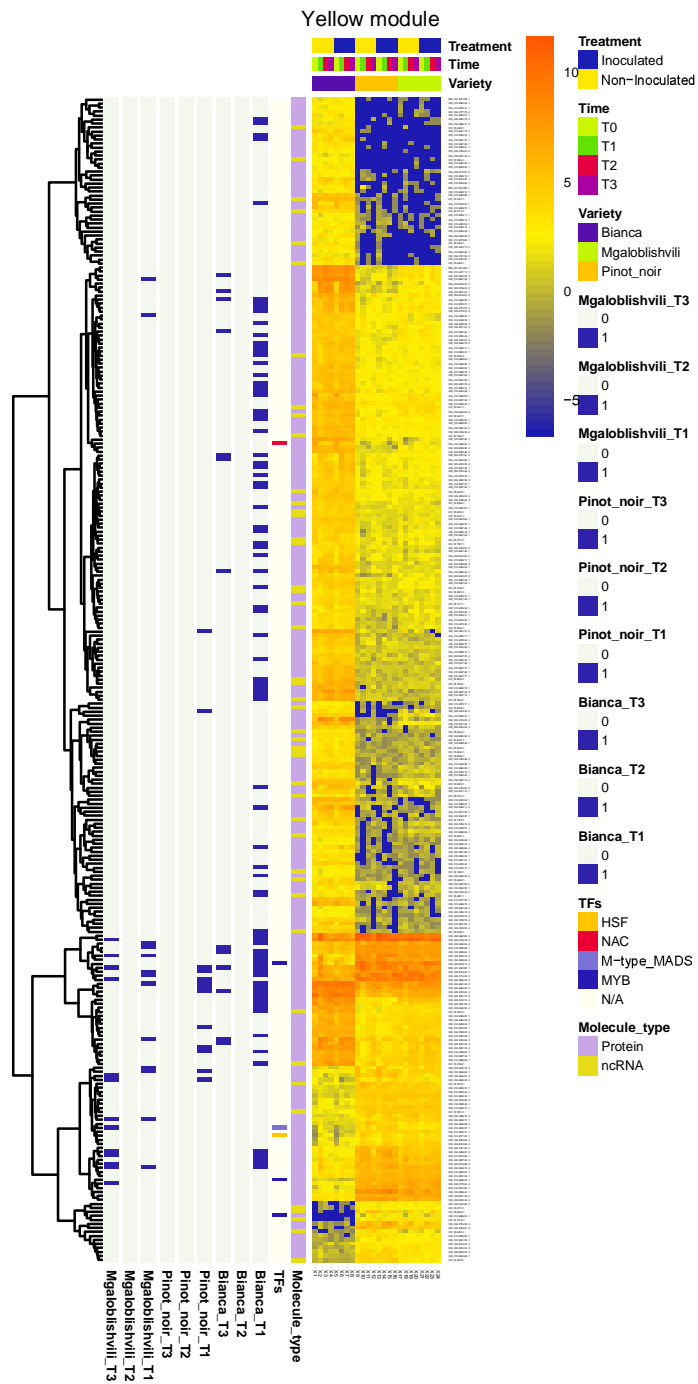

Supplement: Supplementary file 1 [file genes-11-00261-s001.zip › Figure S1.pdf]

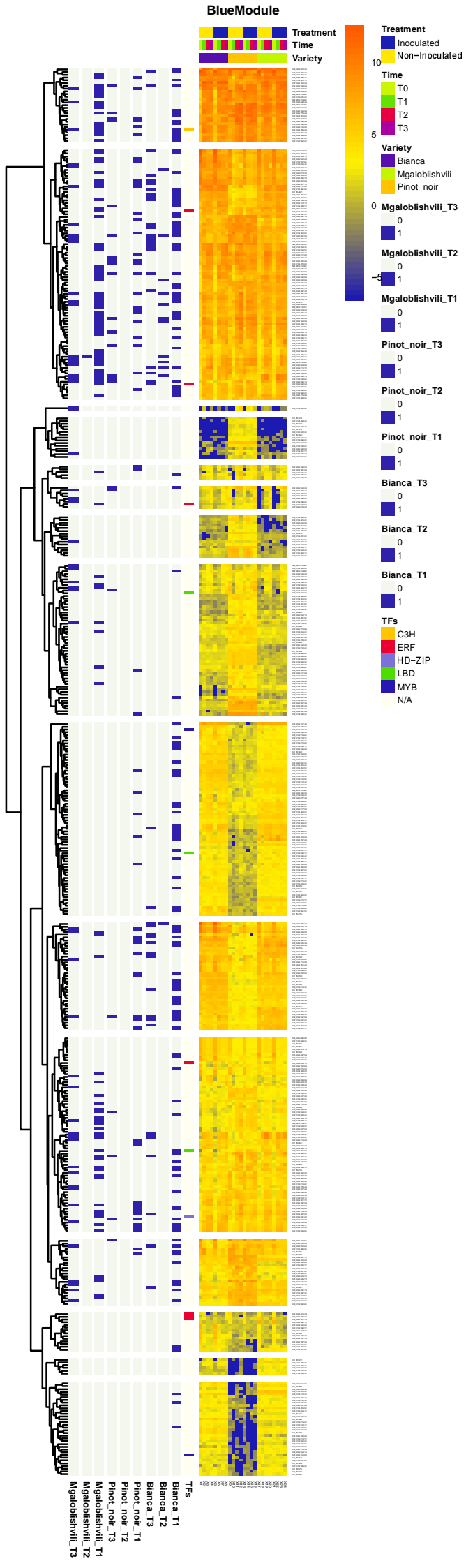

Supplement: Supplementary file 1 [file genes-11-00261-s001.zip › Figure S2.pdf]

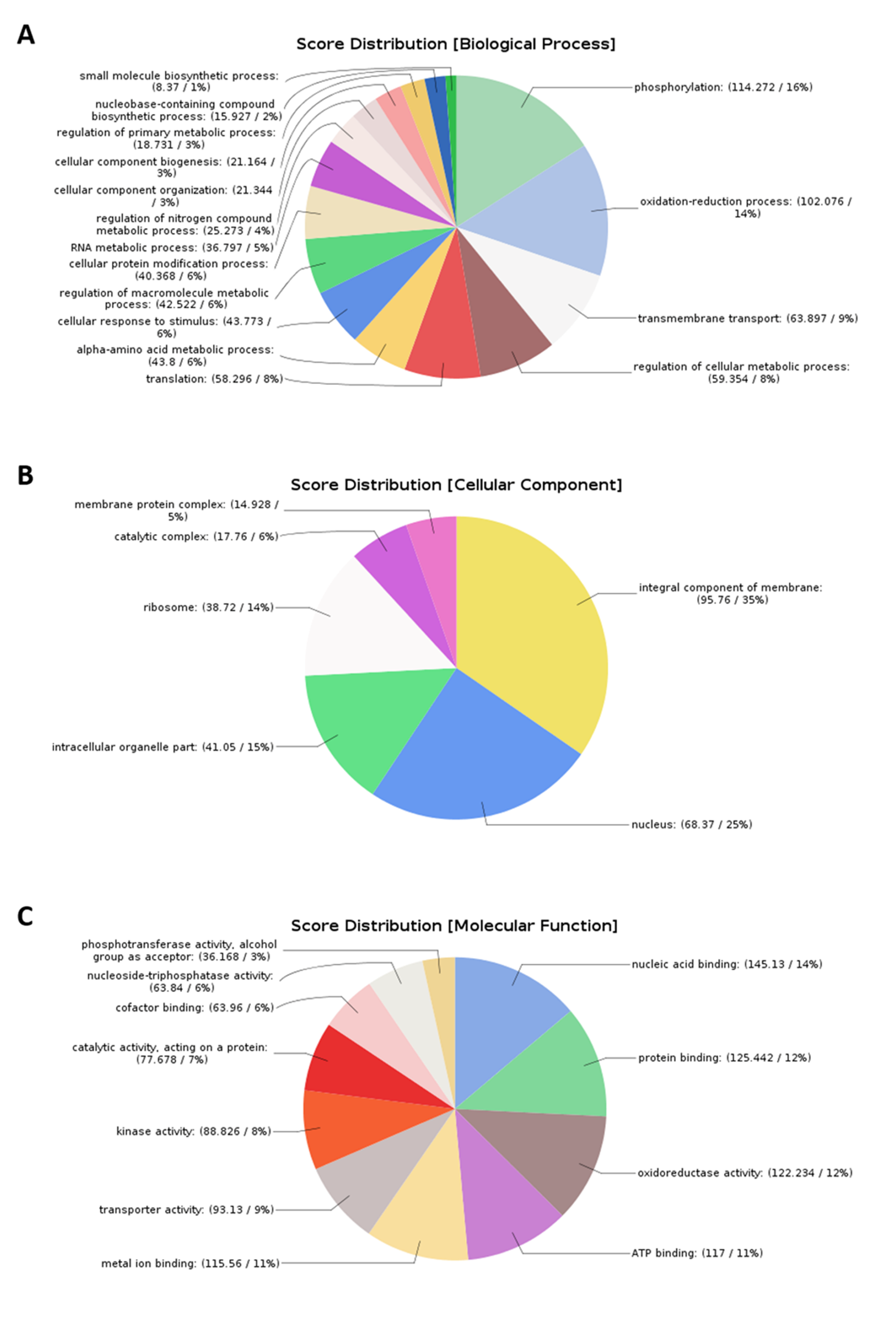

Supplement: Supplementary file 1 [file genes-11-00261-s001.zip › Figure S3.tif]

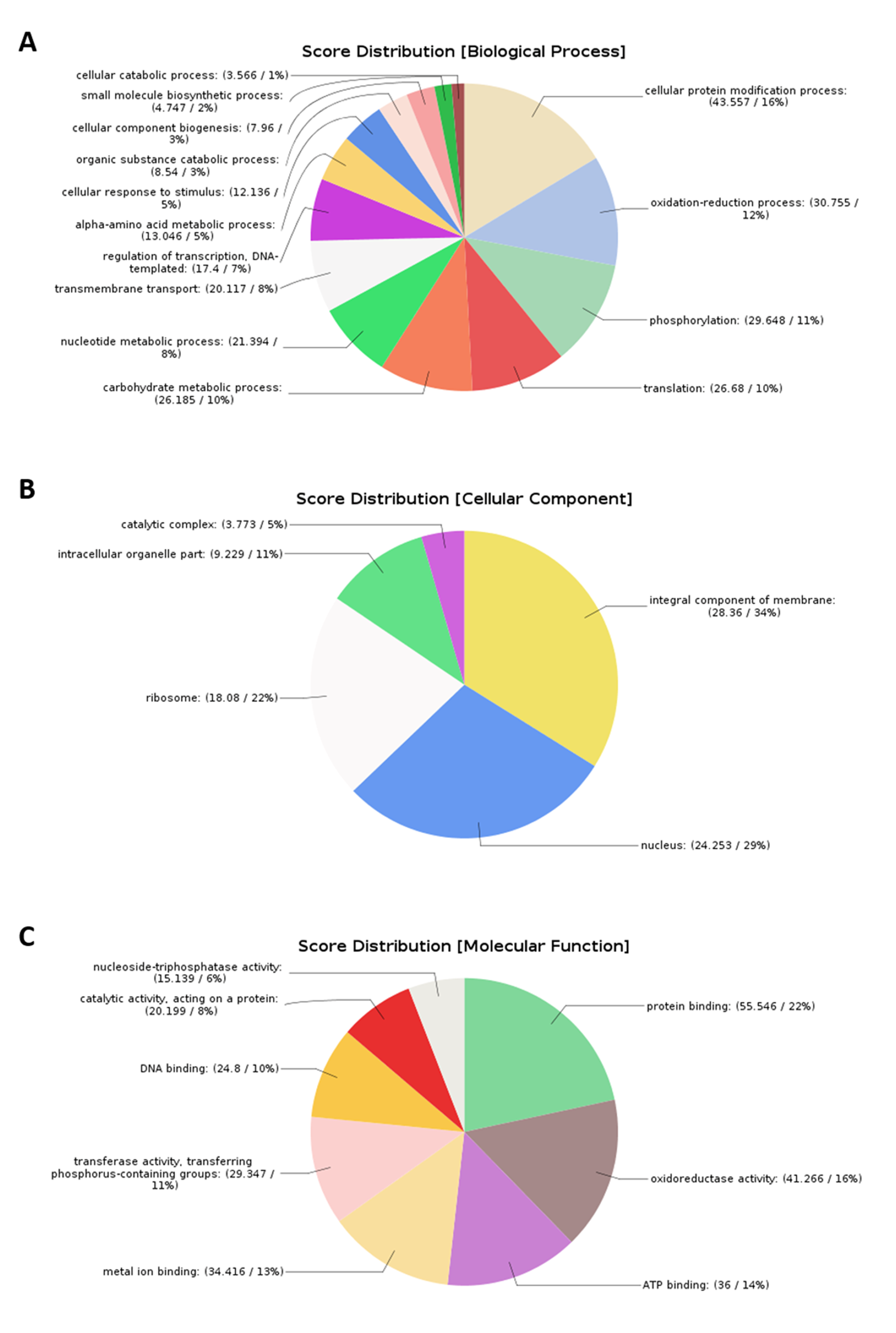

Supplement: Supplementary file 1 [file genes-11-00261-s001.zip › Figure S4.tif]

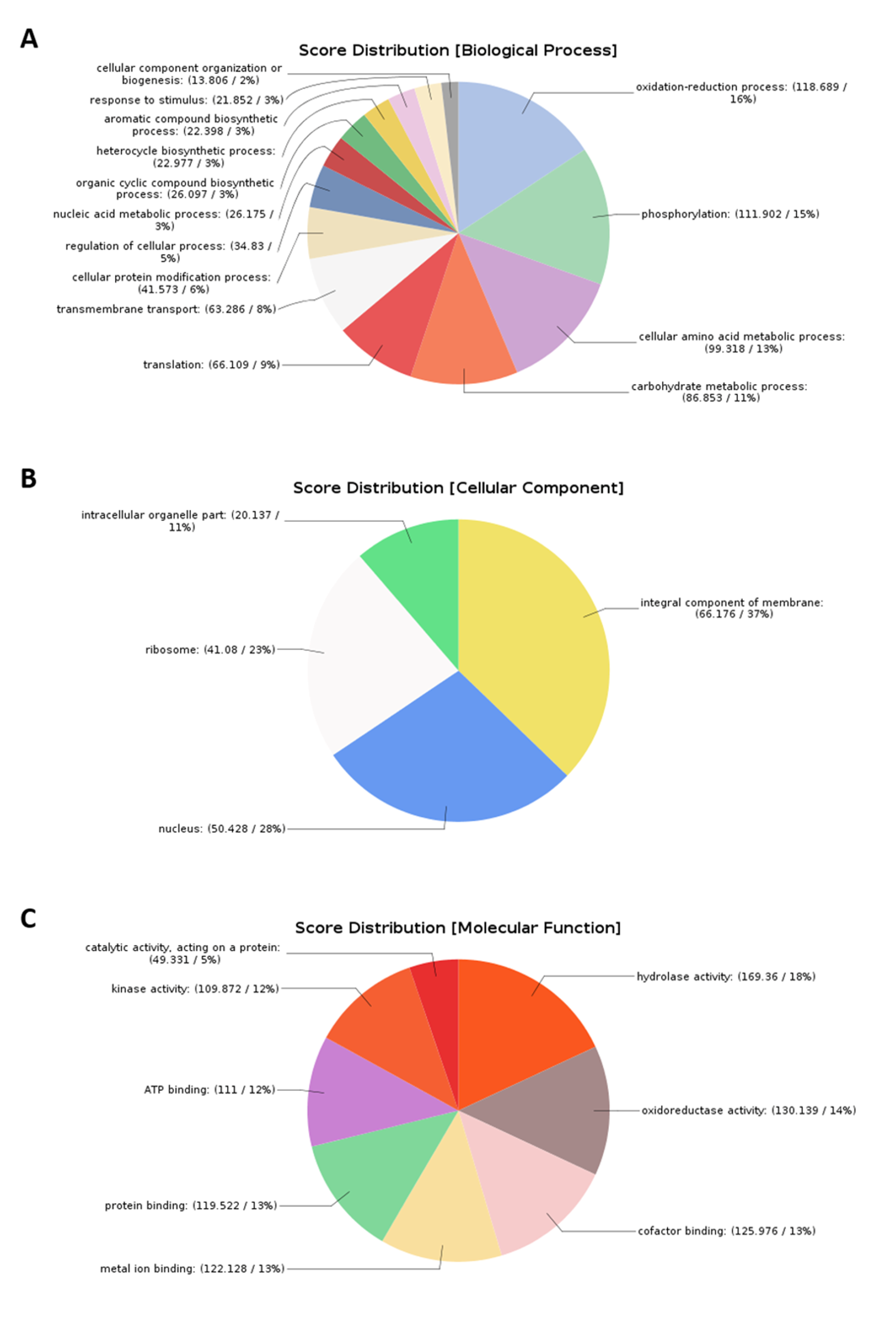

Supplement: Supplementary file 1 [file genes-11-00261-s001.zip › Figure S5.tif]

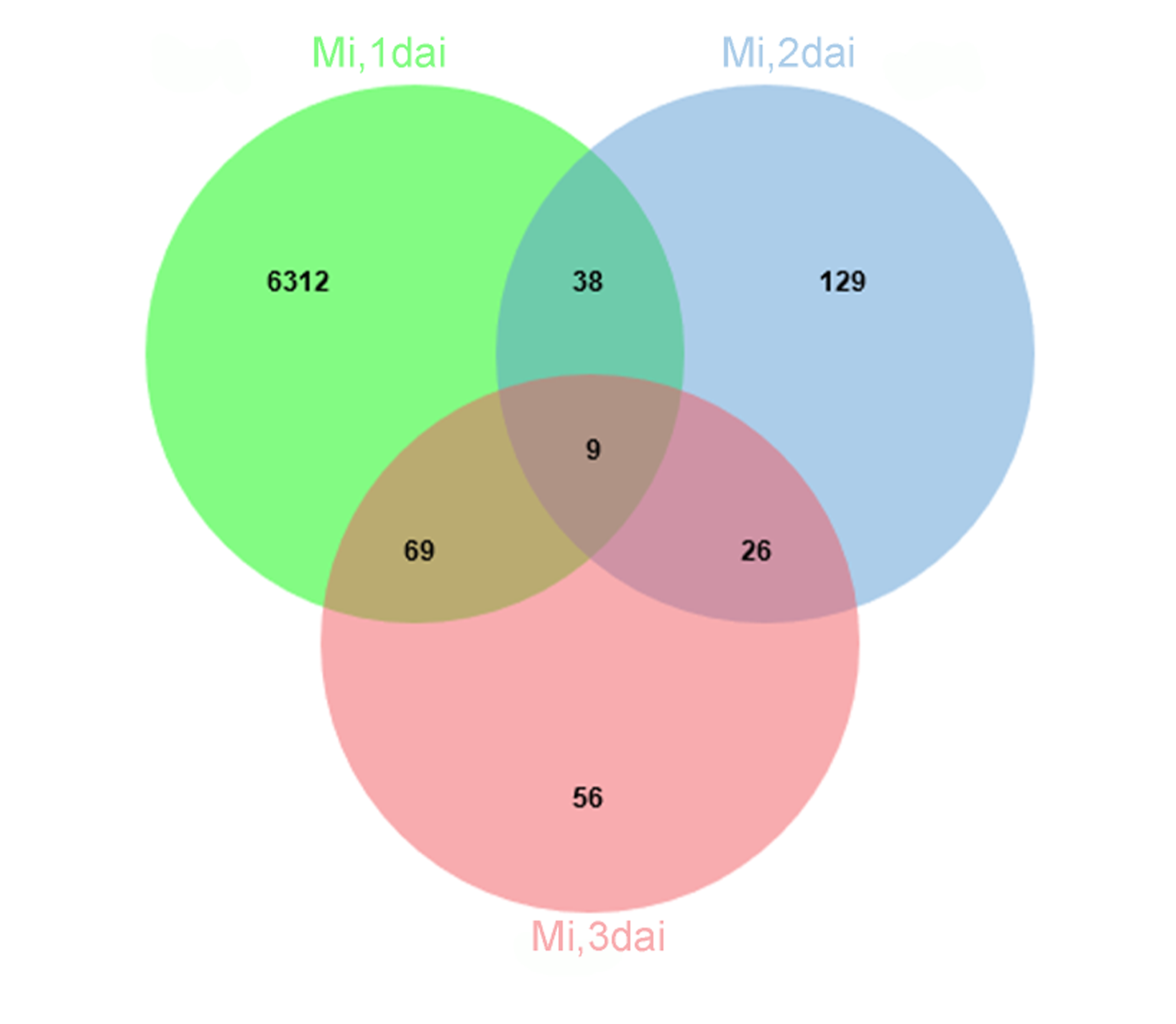

Supplement: Supplementary file 1 [file genes-11-00261-s001.zip › Figure S6.tif]

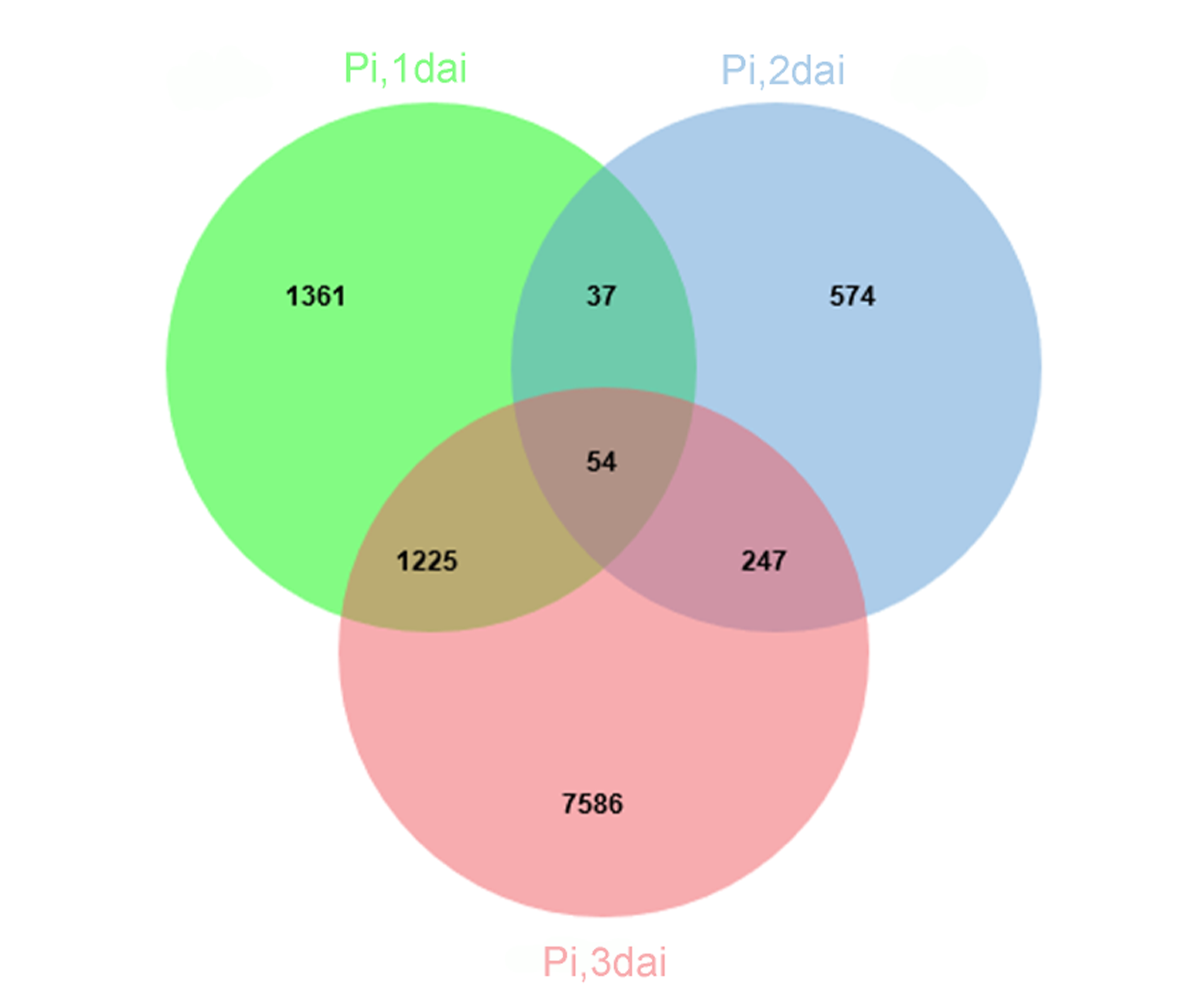

Supplement: Supplementary file 1 [file genes-11-00261-s001.zip › Figure S7.tif]

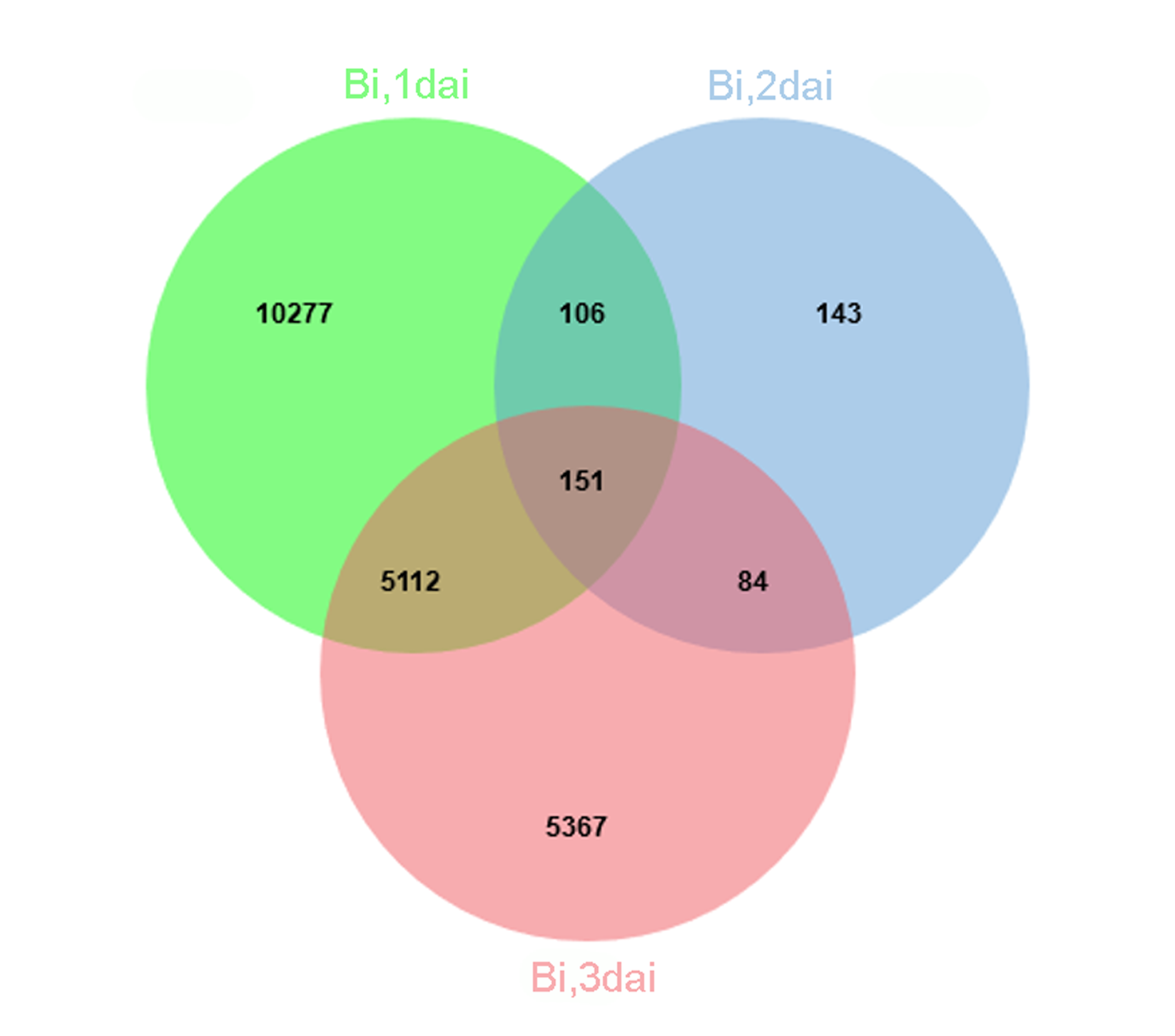

Supplement: Supplementary file 1 [file genes-11-00261-s001.zip › Figure S8.tif]
